# Facile Synthesis of Uranium Complexes with a Pendant Borane Lewis Acid and 1,2‐Insertion of CO into a U−N Bond

**DOI:** 10.1002/anie.202212823

**Published:** 2022-11-17

**Authors:** Wei Su, Thayalan Rajeshkumar, Libo Xiang, Laurent Maron, Qing Ye

**Affiliations:** ^1^ School of Chemistry and Environmental Engineering Anhui Polytechnic University 241000 Wuhu China; ^2^ Department of Chemistry Southern University of Science and Technology 1088 Xueyuan Blvd., Xili, Nanshan District 518055 Shenzhen China; ^3^ Laboratoire de Physique et Chimie des Nanoobjets INSA CNRS UPS Université de Toulouse 31077 Toulouse France; ^4^ Institute for Inorganic Chemistry Julius-Maximilians-Universität Würzburg Am Hubland 97074 Würzburg Germany; ^5^ Institute for Sustainable Chemistry & Catalysis with Boron Julius-Maximilians-Universität Würzburg Am Hubland 97074 Würzburg Germany

**Keywords:** Boranes, Boron, Carbon Monoxide, Lewis Acids, Uranium

## Abstract

In this contribution, we illustrate uranium complexes bearing a pendant borate (i.e. **1** and **2**) or a pendant borane (i.e. **3** and **4**) moiety via reaction of the highly strained uranacycle **I** with various 3‐coordinate boranes. Complexes **3** and **4** represent the first examples of uranium complexes with a pendant borane Lewis acid. Moreover, complex **3** was capable of activation of CO, delivering a new CO activation mode, and an abnormal CO 1,2‐insertion pathway into a U−N bond. The importance of the pendant borane moiety was confirmed by the controlled experiments.

## Graphical abstract

Reactions between the highly strained uranacycle **I** with various boranes afford unprecedented uranium complexes bearing either a pendant borate or a pendant borane Lewis acid. The latter promotes the 1,2‐insertion of CO into a U^IV^‐N bond.

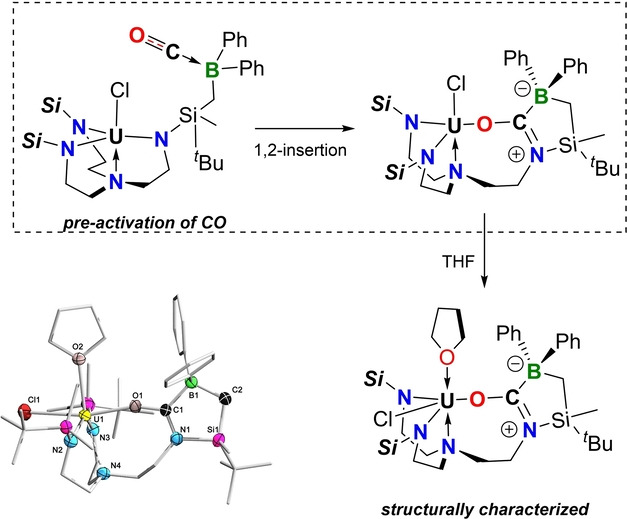

Recent findings about uranium complexes bearing N/P^[1]^ or O/P^[2]^ double‐layer ligands demonstrated the relevance of pendant or secondary‐sphere coordinating sites in terms of the construction of uranium‐transition metal bonds. The secondary binding sites are dominated by the basic phosphino groups that coordinate to the elements, particularly those soft metals spanning the periodic table,^[3]^ thus allowing for the hard‐soft heterometallic bonding that would otherwise not be favored. Moreover, recent reports have opened up the prospect of such heterometallic clusters in small molecule activation, such as N_2_.^[4]^ However, *f*‐metal complexes bearing a pendant Lewis acidic borane moiety serving as the secondary binding site are thus far not reported. The stagnation stands out against the backdrop of the boom in *d*‐metal complexes comprising one or several pendant Lewis acidic borane moieties.^[5]^ For instance, the dangling Ph_2_P(CH_2_)_2_B(C_8_H_14_) of complex **A** is indispensable to further mediate reductive coupling of CO to form a C−C bond in the presence of NaHBEt_3_ (Figure 1a, top).^[6]^ Drover and *co*‐workers demonstrated that the appended borane moiety has great impact on the ability of the hydride complexes [**B**H]^+^ in hydride transfer, as well as the oxidative addition behavior at the Ni^0^ center in **B**.^[7]^ Very recently, Chu, Szymczak and the co‐workers successfully applied a pendant 9‐BBN (borabicyclo[3.3.1]nonyl) group to stabilize the reactive Ni^II^ imido intermediates (Ni=NR) thanks to the presence of the secondary B−N interactions (**C**), and thus achieved the intermolecular 1,2‐C_Ar_‐H‐addition or intramolecular 1,2‐C_benzylic_‐H‐addition across the Ni=N bonds.^[8]^ It is noteworthy that the unique chemical behavior of the *d*‐metal complexes bearing acidic groups in the secondary coordination sphere is commonly associated with the oxidative‐addition/reduction‐elimination process,[^5d^, ^6^, ^7a^, ^7b^, ^8^] which is nevertheless unfavorable for *f*‐metals. As such, uranium complexes with appended borane moieties should exhibit distinctive reactivity profiles, which led us to focus on this class of compounds.

**Figure 1:**
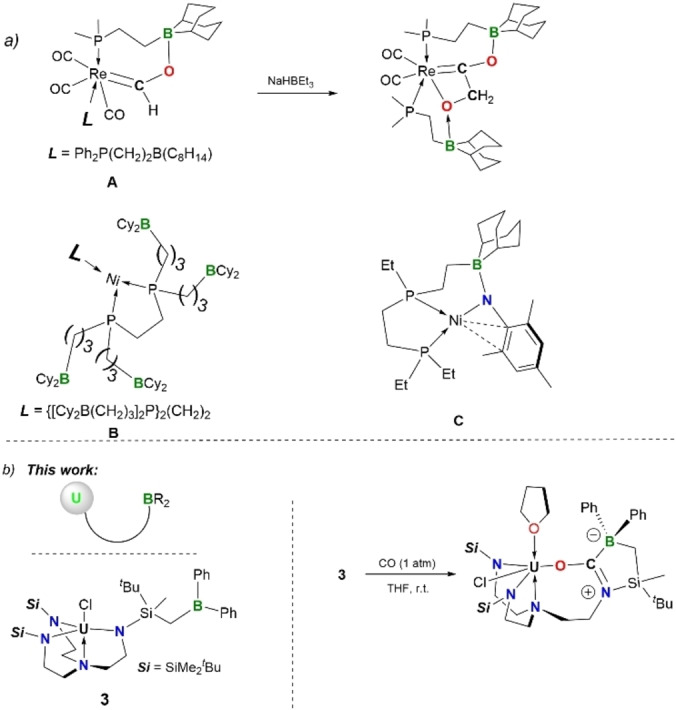
a) Representative examples of *d*‐metal complexes **A**, **B** and **C** with appended borane moieties; b) the uranium complex (**3**) bearing a pendant borane and its activation of CO in this work.

The paucity of the uranium complexes with pendant Lewis acidic borane moieties (Figure 1b, top left) is most likely due to the lack of an experimental synthetic approach. Considering that such molecular system featuring two Lewis acidic centers (i.e. U and B) may have potential in U−B bond construction, small molecule activation and Lewis acid catalysis, we herein set out to synthesize the first uranium complexes comprising a pendant tricoordinate borane Lewis acid.

Initially, reactions of the uranacycle **I** ^[9]^ with tris(pentafluorophenyl)borane B(C_6_F_5_)_3_ and the Piers’ borane^[10]^ HB(C_6_F_5_)_2_ were performed, affording the uranium borate complexes **1** and **2**, respectively (Scheme 1, top). The atom connectivity of both products was confirmed by single crystal X‐ray diffraction analysis (Figure 2). Complexes **1** and **2** crystallized in space group $P\bar{1}$ and *P2_1_/n*, respectively. The single crystal structures clearly indicated in both cases the nucleophilic abstraction of the alkyl group from uranium. The U1−F1 distance in **1** is 2.778(2) Å, which is remarkably longer than the covalent single U−F bonds, but within the sum of Van der Waals radii of U (1.86 Å) and F (1.47 Å) atoms, implying the existence of a U1−F1 interaction. In complex **2**, the uranium and boron centers are bridged by a hydrogen atom with a short U1−H1 distance of 2.288(3) Å and a B1−H1 distance of 1.219(3) Å. Thus, **2** can be viewed as an intramolecular borane capped complex of type U−E→BAr_3_.^[11]^ The average U‐N_amide_ (2.20 Å) and U‐N_amine_ (2.60 Å) distances in **1** and **2** are similar and fall in the expected range for a U^IV^ nucleus. Complexes **1** and **2** were characterized in solution as well. The ^11^B NMR spectrum of **1** (C_6_D_6_) displayed a sharp singlet at *δ*_B_ −38.7, while that of **2** displayed a doublet at δ_B_ −46.8, unambiguously confirming the retention of the B1−H1 bond in **2**. In addition, the ^11^B NMR signals of **1** and **2** are remarkably upfield shifted compared to the free borate species such as [RB(C_6_F_5_)_3_]^−^ (R=Me *δ*_B_ −11.9, Et *δ*_B_ −9.1),^[12]^ which is most likely attributed to the existence of a paramagnetic uranium nucleus. Likewise, the ^1^H NMR spectra displayed resonances in a wide signal window, ranging from +71.66 to −72.20 ppm for **1**, and from +55.82 to −185.22 ppm for **2**. The ^1^H resonance of **2** at −185.22 ppm might be assigned to the bridging hydride. The presence of bridging hydride in **2** is further supported by the IR spectrum of **2** (Figure S22), which displayed an absorption band at 2100 cm^−1^ (computed to be 2230 cm^−1^) for the B−H stretching. This is lower than that (2400–2500 cm^−1^) of the classical B−H bonds, but comparable to that of the monodentate U−H−B unit with a bridging hydride.^[13]^ The interaction between B−H and the uranium center is further highlighted by the Wiberg Bond Indexes (WBI). The U−H WBI is 0.08 in line with a hydrogen bond while the B−H one is 0.88 in line with a mainly covalent interaction. Finally, donation from the B−H onto an empty orbital of uranium is also observed at the second order donor‐acceptor level in the Natural Bonding Orbital (NBO) analysis (see Table S19 for details).

**Scheme 1:**
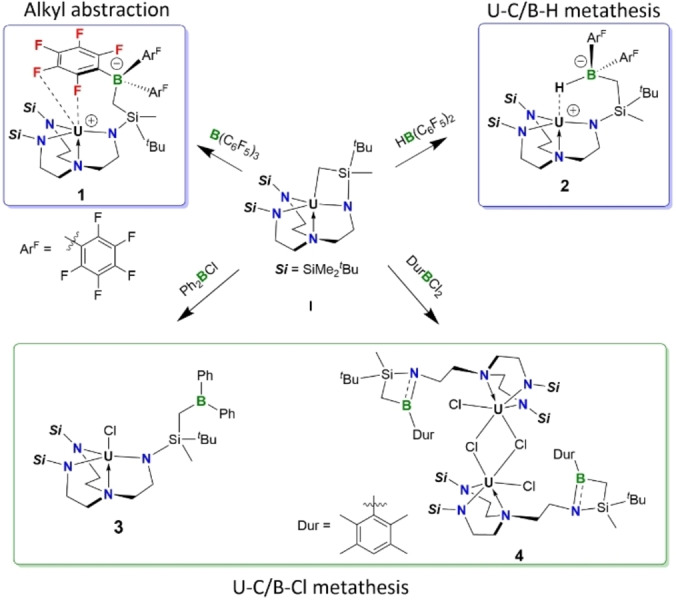
Reaction between uranacycle **I** and various boranes via: alkyl abstraction (**1**), U−C/B−H metathesis (**2**) and U−C/B−Cl metathesis (**3** and **4**).

**Figure 2:**
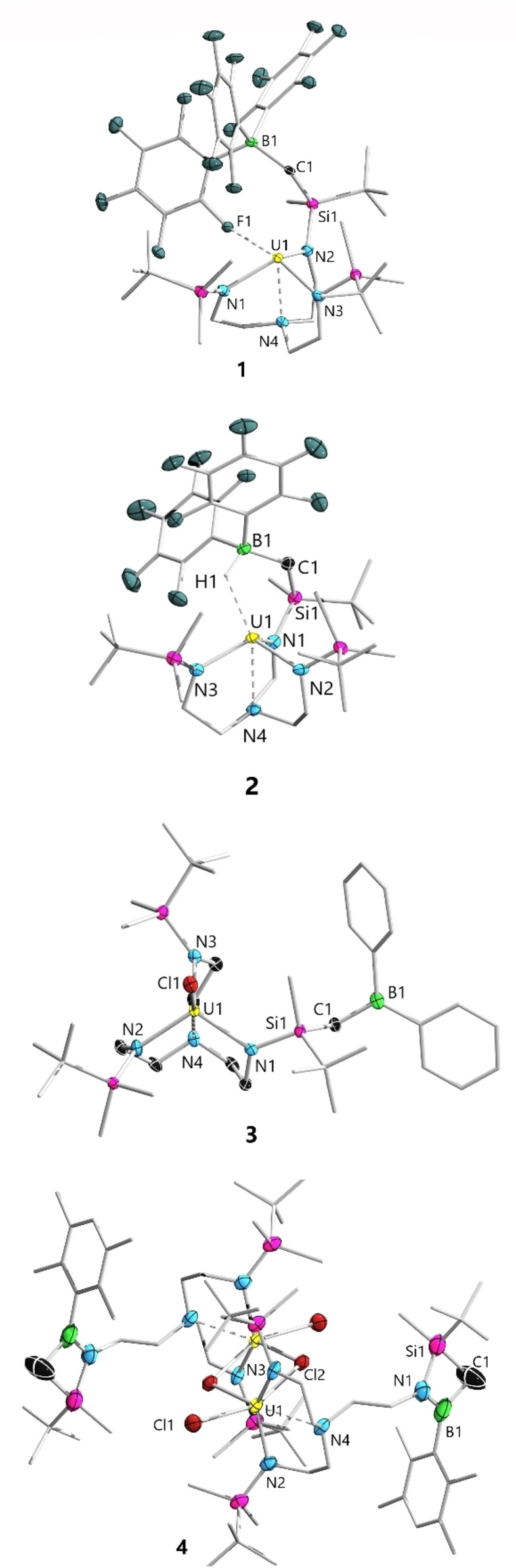
Molecular structures of complex **1**–**4** in the solid state. Solvent molecules and hydrogen atoms were omitted for clarity except the bridging H1 of **2**. Selected bond lengths [Å] and angles [°]: **1**, U1−F1 2.778(2), U1−F2 2.981(2); **2**, U1−H1 2.288, B1−H1 1.219; **3**, U1−Cl1 2.6761(8), B1−C1 1.558(5), U1−N_amide_ 2.20, U1−N4 2.696(3); **4**, U1−Cl1 2.5997(15), U1−Cl2 2.8204(15), B1−N1 1.383(9), N1−Si1 1.810(7), B1−C1 1.616(11), C1−Si1 1.907(9), B1−Si1 2.324(12), N1−B1−C1 103.1(7), N1−Si1−C1 79.0(3), Si1−N1−B1 93.2(5), B1−C1−Si1 81.6(4).

The target complex comprising a pendant tricoordinate borane function would be attained if the uranium nucleus in **2** could abstract the hydride and completely cleave the BH bond. However, all attempts in this regard failed as indicated by the resistant ^11^B doublet signal when [HNEt_3_][BPh_4_] was applied as a hydride abstraction reagent. The treatment of **2** with [Ph_3_C][B(C_6_F_5_)_4_] as a hydride abstraction reagent merely led to unidentified mixtures. Therefore, we decided to replace the hydride of the Piers’ borane with a chloride (i.e. utilize Ar_2_BCl instead). This was mainly based on the consideration that the chlorophilic nature^[14]^ of uranium might facilitate the final chloride abstraction step to give the target complex. Gratifyingly, the reaction of **I** with Ph_2_BCl yielded **3**, which was in full agreement with our hypothesis. In solution, complex **3** showed a broad resonance at *δ*_B_ 83.9, which is comparable to that of Ph_2_BMe (*δ*_B_ 70.6)^[15]^ and an isolable uranium metallaboracycle (*δ*_B_ 76.0).^[16]^ The single crystal structure of **3** revealed a dangling trigonal planar Ph_2_B(alkyl) moiety as indicated by the sum of angles (360.0°) around the boron center. The trigonal bipyramidal geometry around the uranium nucleus in **3** resembles its precursor **I**.^[17]^

We also tested whether the synthetic protocol could be applied to the synthesis of uranium complexes with a pendant haloborane Lewis acid. To this end, complex **1** was reacted with the aryl(dihalo)borane DurBCl_2_ (Dur=2,3,5,6‐tetramethylphenyl) under identical conditions, which nevertheless yielded a dinuclear uranium complex **4** as suggested by single crystal X‐ray diffraction analysis. Indeed, the formation of **4** corresponds to an identical U−C/B−Cl metathesis reaction that proceeds with Ph_2_BCl, which is nevertheless followed by a further U−N/B−Cl metathesis step due to the presence of the second B−Cl bond in DurBCl_2_. Thus, **4** represents another example of the title complexes, yet with the pendant borane function existing in the form of a NBSiC four‐membered ring.^[18]^ The borane function in **4** should be less Lewis acidic than that in **3** due to the presence of considerable N→B π interaction as indicated by the short BN bond of 1.383(9) Å. In the solution state, **4** displayed a pair of doublets at *δ*_H_ −7.91 (*J*=15.7 Hz) and −8.52 (*J*=14.6 Hz) for the geminal methylene protons BC*H*_2_Si.

The successful synthesis and characterization of **3** allowed further investigation of its reaction chemistry. In view of the oxophilic nature of uranium and the tendency of CO to interact with Lewis acidic tricoordinate boron with its carbon end,^[19]^ CO should be a rather promising candidate. Firstly, we decided to investigate the reaction of an analogous uranium complex but without the pedant borane function. To this end, U(Trens^DMTB^)Cl (**6**) was exposed to 1 atm CO (Scheme 2, top) and no reaction was observed by ^1^H NMR monitoring at ambient or elevated temperature (See Supporting Information for details). In fact, this finding was not surprising as only a handful of examples of successful CO insertion into a U^IV^‐N bond are documented in the literature.^[20]^ Next, the title complex **3** was exposed to CO under the same reaction conditions (Scheme 2, bottom). In stark contrast, the formation of a new boron‐containing product was clearly indicated by the ^11^B NMR spectrum, which displayed a new upfield shifted singlet at −3.5 ppm.

**Scheme 2:**
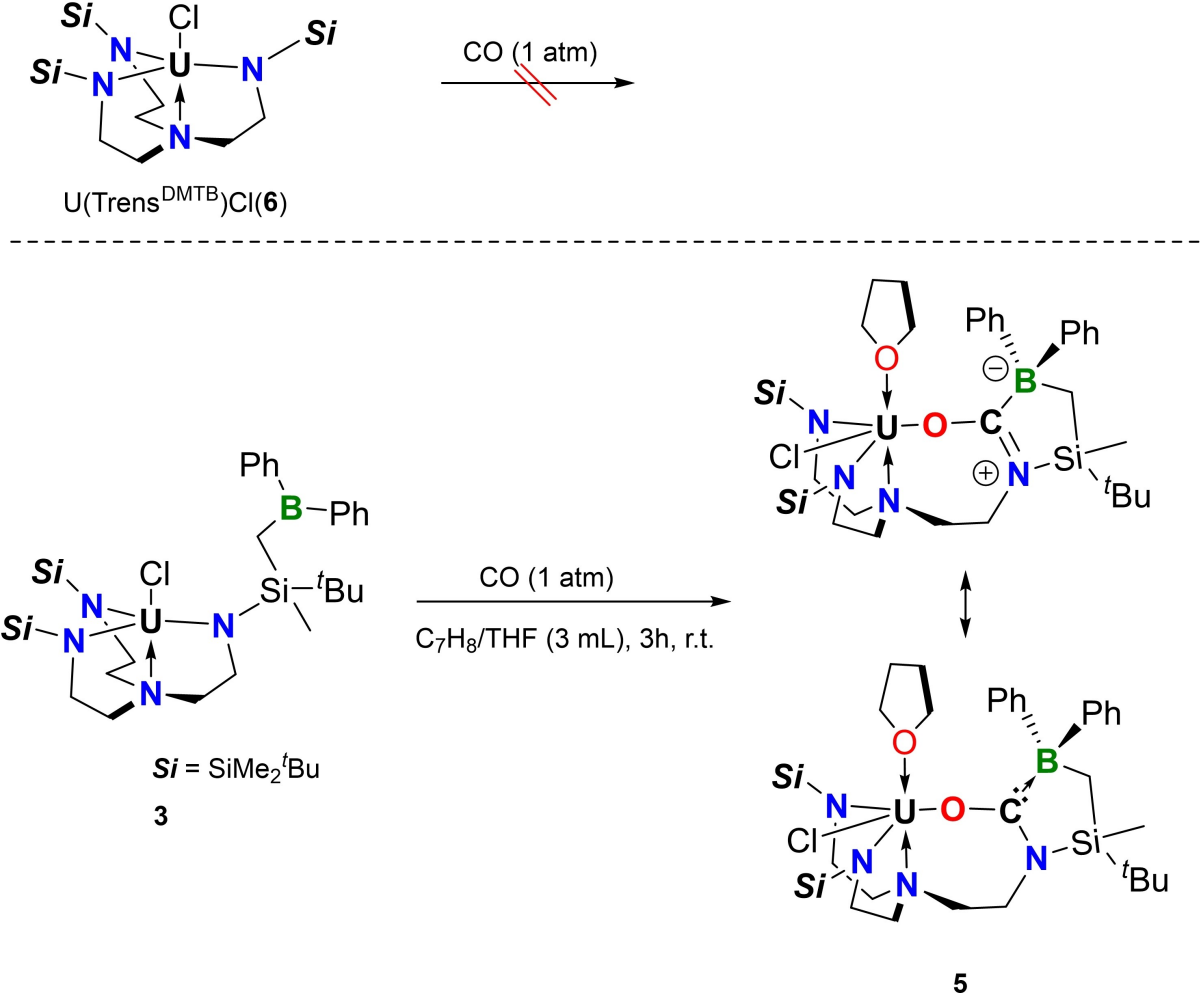
Activation of CO by **3** and the controlled experiment with **6**.

After workup, the product was isolated as a green crystalline solid suitable for single crystal X‐ray diffraction analysis, which indicated the atom connectivity of **5** (Figure 3). Indeed, the formation of **5** represents a rare example of 1,2‐insertion of CO into a U−N bond in the way that the O atom was attached to the oxophilic uranium center and the corresponding (oxy)(amino) carbene (U−O−C(:)−N) was trapped by the Lewis acidic borane function. The C−O bond of 1.297(7) Å is significantly elongated in comparison to the CO triple (1.13 Å) and C=O double bonds (1.21 Å). The C1−N1 bond length of 1.329(7) Å is in line with a typical C=N double bond. The U1−O1 bond length of 2.258(4) Å is comparable to the typical U−O single bonds,^[21]^ but remarkably shorter than that of a U−O dative bond (i.e. U1<O2 in **5**, 2.472(4) Å). The IR spectrum of **5** displays absorption bands at 1590 and 1180 cm^−1^, which are assigned to C−O and C−N bond, respectively. The decrease in C−N stretching frequencies in comparison to typical C=N double bonds can be rationalized by the contribution from the carbene‐type form (bottom right of Scheme 2).

**Figure 3:**
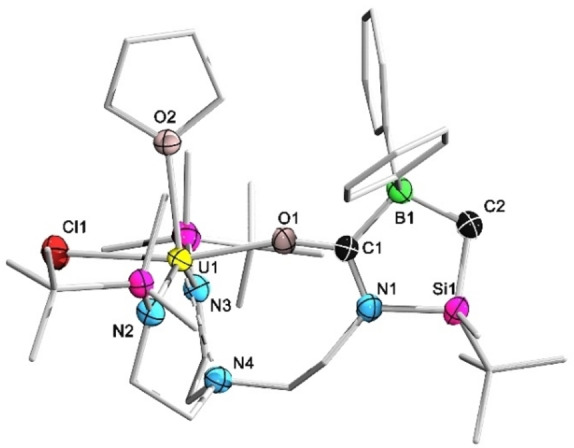
Molecular structures of **5** in the solid state. Hydrogen atoms are omitted for clarity. Selected bond lengths [Å] and angles [°]: U1−O1 2.258(4), C1−O1 1.297(7), C1−N1 1.329(7), C1−B1 1.655(9), U1−Cl1 2.6562(16), U1−O2 2.472(4), U1−N4 2.612(5), U1−N2/N3 2.216(5)/2.203(8), C2−B1 1.658(9), C1−O1−U1 160.6(4), C2−Si1−N1 96.0(3).

The formal oxidation states of uranium core in these complexes were verified by variable‐temperature (2–300 K) SQUID magnetization measurements (Figure 4). Complexes **1**–**5** possess RT magnetic moments (per ion) of 2.51, 3.14, 2.86, 3.42, 2.83 μ_B_, respectively, which decrease to 0.72, 0.63, 0.49, 1.05, 0.41 μ_B_ at 2 K, respectively. These data are consistent with the presence of 5*f* ^2^ U^IV^ centers in these complexes.^[22]^

**Figure 4:**
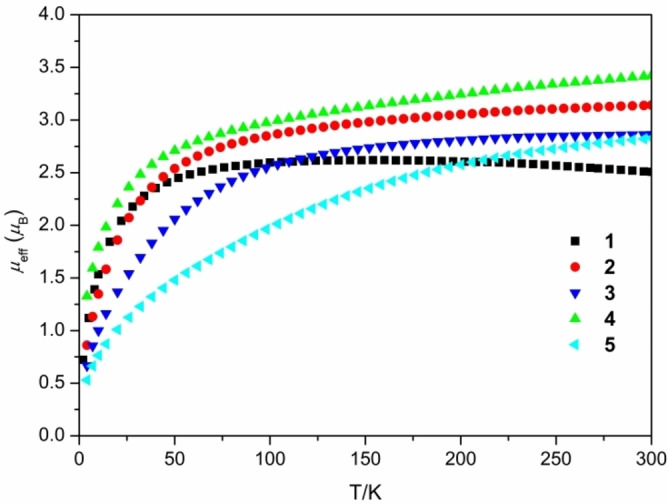
Temperature‐dependent SQUID magnetization for **1**–**5** at 1000 Oe.

To get some insights on the activation of CO by complex **3**, DFT calculations were carried out on the reaction mechanism (Figure 5). The reaction is a two‐step reaction where the CO molecule first binds the boron and then inserts into the U−N bond.

**Figure 5:**
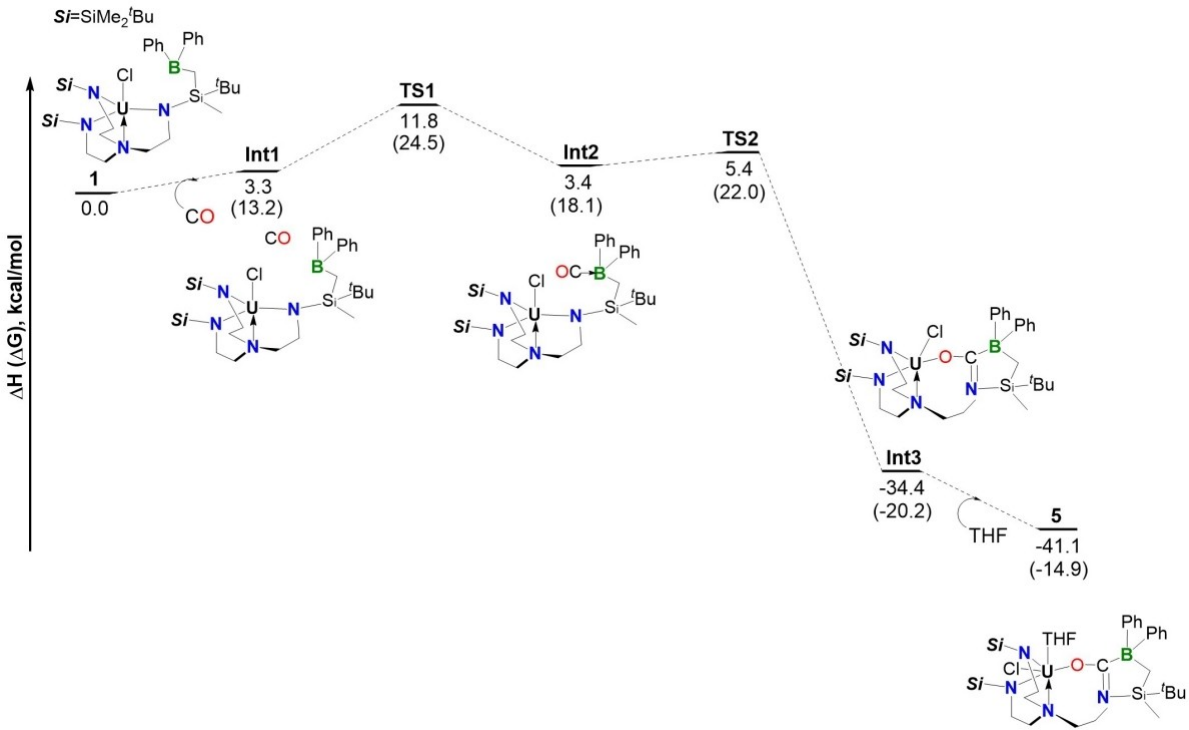
Computed reaction profile at room temperature in kcal mol^−1^. The enthalpy is reported and the Gibbs Free energy are given between bracket.

The CO binding to boron transition state (TS) was located on the Potential Energy Surface (PES) and the associated barrier is relatively low 11.8 kcal mol^−1^. At the TS, the B−C bond is not yet formed (2.20 Å) and the CO bond is slightly elongated (1.13 Å). Therefore, the CO binding is activating the bond as further evidenced by the Wiberg bond index (WBI) at the TS that is 2.27 instead of 3.00 for free CO. From **TS1**, the system evolves to form a CO adduct (**Int2**) whose formation is slightly endothermic (3.4 kcal mol^−1^). However, since the CO bond is activated, the system further reacts through **TS2**. The associated barrier is 5.4 kcal mol^−1^ (2.0 kcal mol^−1^ from **Int2**) in line with a kinetically facile almost one‐step reaction. At **TS2**, neither the C−N (2.39 Å) nor the U−O bonds (3.74 Å) are formed. The CO bond is slightly elongated (1.15 Å) so that the electron density is not yet relocalized. Following the intrinsic reaction coordinates, it yields the formation of a THF free form of complex **Int3**, whose formation is thermodynamically favorable (−34.4 kcal mol^−1^). Finally, the coordination of a THF molecule further stabilizes the formation of complex **Int3** to give **5** (−41.1 kcal mol^−1^) in line with the experimental observation.

In summary, we explored the reactions between the uranacycle alkyl complex **I** with various boranes. While these reactions employing Pier's borane and B(C_6_F_5_)_3_ generate uranium complexes bearing appended borates, unprecedented uranium complexes comprising a pendant borane unit (i.e. **3** and **4**) are isolated when applying arylchloroboranes. The reaction of complex **3** with CO represents a rare example of 1,2‐insertion of CO into a U−N bond. In stark contrast, complex **6** bearing no pendant borane Lewis acid is unreactive with CO, thus highlighting the importance of the pendant borane unit in the secondary coordination sphere, which could be further confirmed by DFT calculations. Indeed, the CO molecule should be pre‐activated by the borane Lewis acid upon OC→B interaction, which lowers the energy required for the CO insertion step. Further studies on the U/B system in terms of activation of more challenging small molecules are currently underway in our laboratory.

## Conflict of interest

The authors declare no conflict of interest.

## Supporting information

As a service to our authors and readers, this journal provides supporting information supplied by the authors. Such materials are peer reviewed and may be re‐organized for online delivery, but are not copy‐edited or typeset. Technical support issues arising from supporting information (other than missing files) should be addressed to the authors.

Supporting Information

Supporting Information

## Data Availability

The data that support the findings of this study are available in the Supporting Information of this article.
