# Supplementary material for: Facile Synthesis of Uranium Complexes with a Pendant Borane Lewis Acid and 1,2‐Insertion of CO into a U−N Bond
Source: Angew Chem Int Ed Engl. 2022 Nov 17;61(51):e202212823. doi: 10.1002/anie.202212823 (PMC10099876; doi:10.1002/anie.202212823)
Supplement: Supplementary file 1 — Supporting Information [file ANIE-61-0-s002.pdf]

Angewandte Chemie
Eine Zeitschrift der Gesellschaft Deutscher Chemiker

Supporting Information

Facile Synthesis of Uranium Complexes with a Pendant Borane Lewis Acid and 1,2-Insertion of CO into a U–N Bond

W. Su, T. Rajeshkumar, L. Xiang, L. Maron, Q. Ye**

Table of Contents

General remarks.. 2

Synthesis and characterizations 3

NMR Spectroscopy.. 6

Magnetic properties 14

Infrared spectroscopy 17

Single-crystal XRD 19

Computational details.. 30

References 72

General remarks.

All the manipulations were carried out using standard schlenk lines or glovebox under an argon atmosphere. All the solvents were dried following standard techniques. Deuterated benzene was distilled from Na/K and stored under an argon atmosphere prior to use. Other reagents were used as received without further purification. The nuclear magnetic resonance spectroscopy was recorded on a Bruker AVIII-400 (^{1}H 400.1 MHz; ^{11}B 128.4 MHz; ^{19}F 376.5 MHz) spectrometer at room temperature. ^{1}H and ^{29}Si NMR spectra were referenced to external TMS**,** ^{11}B and $^{19}F\{^{1}H\}$ NMR spectra were referenced relative to 15% $BF_3{\cdot}OEt_2$**.** Elemental analyses (C, H, N) were performed on a Vario Micro Cube elemental analyser at Shanghai Institute of Organic Chemistry, the Chinese Academy of Sciences. Magnetic properties were measured on MPMS SQUID VSM (Quantum Design). FTIR were measured with an ALPHA II compact FT-IR spectrometer placed in a glovebox filled with Argon. Complex **I** [1], pier's borane $HB(C_6F_5)_2$ [2], $DurBCl_2$ [3] and Ph_2BCl [4] were prepared according to published methods.

*A note on ^{13}C and ^{29}Si NMR spectroscopy: no meaningful spectra could be acquired except for the ^{29}Si NMR spectrum of **3**. This is attributable to the paramagnetic nature of the samples.*

Caution! *Depleted uranium (primary isotope ^{238}U) is a weak α-emitter; manipulations and reactions should be carried out in monitored fume hoods or in an inert atmosphere glovebox in a radiation laboratory equipped with α- and β-counting equipment.*

Synthesis and characterizations

General synthesis： To an orange solution of **I** (144.6 mg, 0.2 mmol) in pentane or hexane (5 mL) was added boranes (0.2 mmol). The mixture was stirred at ambient temperature for 4hrs before work-up. The solution was consequently filtrated, and the filtrate concentrated and stored at -30 ℃ to induce crystallization (for **1**, **2** and **3**). For **4**, a green suspension formed. The suspension was filtered through glass frits and the green residue was washed thoroughly with pentane (3 mL×3) and then redissolved by C_6H_6. Green crystals suitable for SC-XRD analysis were attained by slow evaporation a benzene solution of **4** at ambient temp for 2 days.

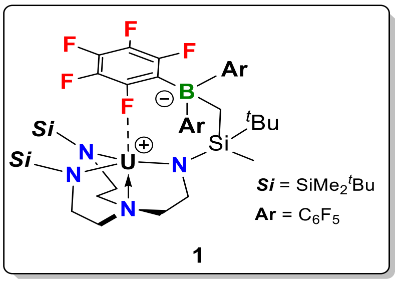

1: Green powder. Yield: 148 mg, 59 %.

^{1}H NMR (C_6D_6): δ 71.66 (1H), 49.82 (1H), 45.16 (1H), 31.34 (9H, ^{t}Bu), 30.15 (3H, Me), 25.58 (3H, Me), 20.11(9H, ^{t}Bu), 14.83 (3H), 4.61 (1H), 3.10 (1H), 1.52 (9H, ^{t}Bu), -7.52 (1H), -18.25 (3H, Me), -26.59 (1H), -36.39 (1H), -38.23 (1H), -38.70 (3H, Me), -46.58 (1H), -47.55 (1H), -56.25 (1H), -72.20 (1H) ppm.

^{11}B NMR (C_6D_6): δ -38.7 ppm.

^{19}F{^{1}H} NMR (C_6D_6): δ -164.7 (br), -168.3 (br) ppm.

Anal. calcd. for $C_{42}H_{56}BF_{10}N_4Si_3U$●$0.25C_6H_{14}$: C, 41.58; H, 4.77; N, 4.46. found: C, 41.84; H, 4.86; N, 4.45.

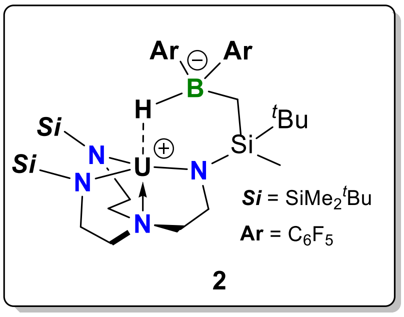

2: Yellowish-green powder. Yield: 107 mg, 51 %.

^{1}H NMR (C_6D_6): δ 55.82 (1H), 47.15 (1H), 43.20 (1H), 33.13 (1H), 30.73 (3H, Me), 29.53 (9H, ^{t}Bu), 23.85 (1H), 20.80 (3H, Me), 17.26 (3H), 16.95 (12H), 14.89 (1H), 1.58 (9H, ^{t}Bu), -1.84 (1H), -4.64 (1H), -18.70 (1H), -35.56 (1H), -36.63 (3H, Me), -39.68 (1H), -46.31 (1H), -48.20 (1H), -73.99 (1H), -185.22 (1H, U***H***B) ppm.

^{11}B NMR (C_6D_6): δ -46.8 (d, J = 43.0 Hz) ppm.

$^{19}F\{^1H\}$ NMR (C_6D_6): δ -163.1(t, J = 17.04 Hz), -167.3 (t, J = 17.91 Hz), -169.0 (br), -175.1 (t, J = 15.04 Hz), -182.1 (br) ppm.

Anal. calcd. for $C_{36}H_{57}BF_{10}N_4Si_3U$: C, 40.45; H, 5.37; N, 5.24. found: C, 40.55; H, 5.46; N, 5.25.

3: Yellowish-green powder. Yield: 120 mg, 65%.

^1H NMR (C_6D_6): δ 29.15 (br, 1H), 11.32 (4H), 10.79 (2H), 10.53 (1H), 8.26 (3H, Me), 7.95 (3H, Me), 7.62 (9H, ^{t}Bu), 7.57 (3H, Me), 7.44 (9H, ^{t}Bu), 6.67 (1H), 6.52 (3H, Me), 6.17 (1H), 5.58 (9H, ^{t}Bu), 5.23 (3H, Me), 4.99 (1H), 4.21 (3H, Me), 3.49 (4H), -23.73 (1H), -25.99 (1H), -26.17 (1H), -26.21 (1H), -26.80 (1H), -27.06 (1H) ppm.

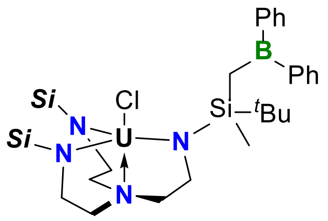

3

^{11}B NMR (C_6D_6): δ 83.9 (br) ppm.

^{29}Si NMR (C_6D_6): δ -27.1 (s), -38.8 (s) ppm.

Anal. calcd. for $C_{36}H_{66}BClN_4Si_3U$: C, 46.82; H, 7.20; N, 6.07. found: C, 46.74; H, 7.44; N, 6.14

4: Green blocks. Yield: 114 mg. 58%.

^1H NMR (C_6D_6): δ 90.95 (3H, Me), 41.83 (6H, Me × 2), 29.96 (18H, ^{t}Bu × 2), 26.02 (6H, Me × 2), 6.75 (1H), 3.73 (3H, Me),1.41 (1H), 0.07 (3H, Me), -4.81 (3H, Me), -5.36 (10H, ^{t}Bu+CH_2), -7.22 (3H, Me), -7.91 (d, J = 15.7 Hz), -8.52(d, J = 14.6 Hz), -16.85 (3H, Me), -33.19 (2H, CH_2), -50.15 (1H), -53.19 (1H), -99.23 (1H), -105.82 (1H) ppm.

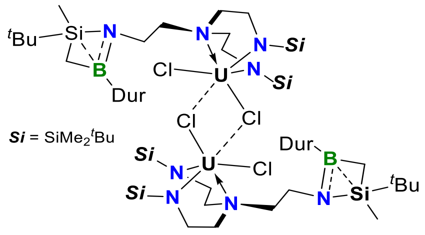

4

^{11}B resonance of **4** was not detected, presumably owing to the paramagnetic U(IV) center. As a reference: our recent publication illustrated a similar four-membered ring, exhibiting a ^{11}B resonance at 41.4 ppm.[5]

Anal. calcd. for $C_{68}H_{138}B_2Cl_4N_8Si_6U_2•C_6H_6$: C, 45.49; H, 7.43; N, 5.73. found: C, 45.57; H, 7.60; N, 5.67.

Activation of CO by 3:

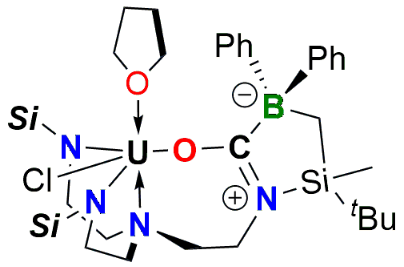

Synthesis of **5**: A green solution of **3** (200 mg, 0.22 mmol) in toluene-THF (v/v = 4/1,3 ml) was "frozen-pump-thaw" for three times and then CO was filled at ambient temperature. The green solution immediately turned to dark brown upon exposure to CO. The mixture was stirred at ambient temperature for 3 h. Consequently, solvent was removed under dynamic vacuum, giving brown residue. The residue was washed with hexane (5 mL*3), affording crude **5** as pale green powders. Crystals suitable for XRD analysis were attained by dissolving the pale green powders with toluene/hexane (1/1) and storing the saturated toluene/hexane solution at -30 ^{o}C overnight. The ca. 15 mL brown hexane filtrate obtained from washing was collected and stored at -30 ^{o}C to afford another batch of crystalline **5**. Those crystals are collected. Yield: 105 mg, 50%.

NOTE: Do not prolong the reaction time. Otherwise, the gradual formation of unidentified by-products (δ^{11}B -13.5 and -22.6) could be observed, which will lead to difficult in crystallization of **5** from the mixture.

^{1}H NMR (C_6D_6): δ 49.36, 43.61, 32.52, 20.12, 19.25, 16.82, 14.92, 12.04, 8.82, 7.41, 4.99, 2.81, 0.15, 0.15, -1.42, -12.96, -16.01, -20.27, -22.21, -29.66 -38.43, -45.69, -50.65, -58.03, -94.31 ppm.

^{11}B NMR (C_6D_6): δ -3.5 (s) ppm.

Anal. calcd. for $C_{41}H_{74}BClN_4Si_3UO_2$•$0.5C_6H_{14}$: C, 49.54; H, 7.65; N, 5.25. found: C, 49.44; H, 7.42; N, 5.43.

NMR Spectroscopy

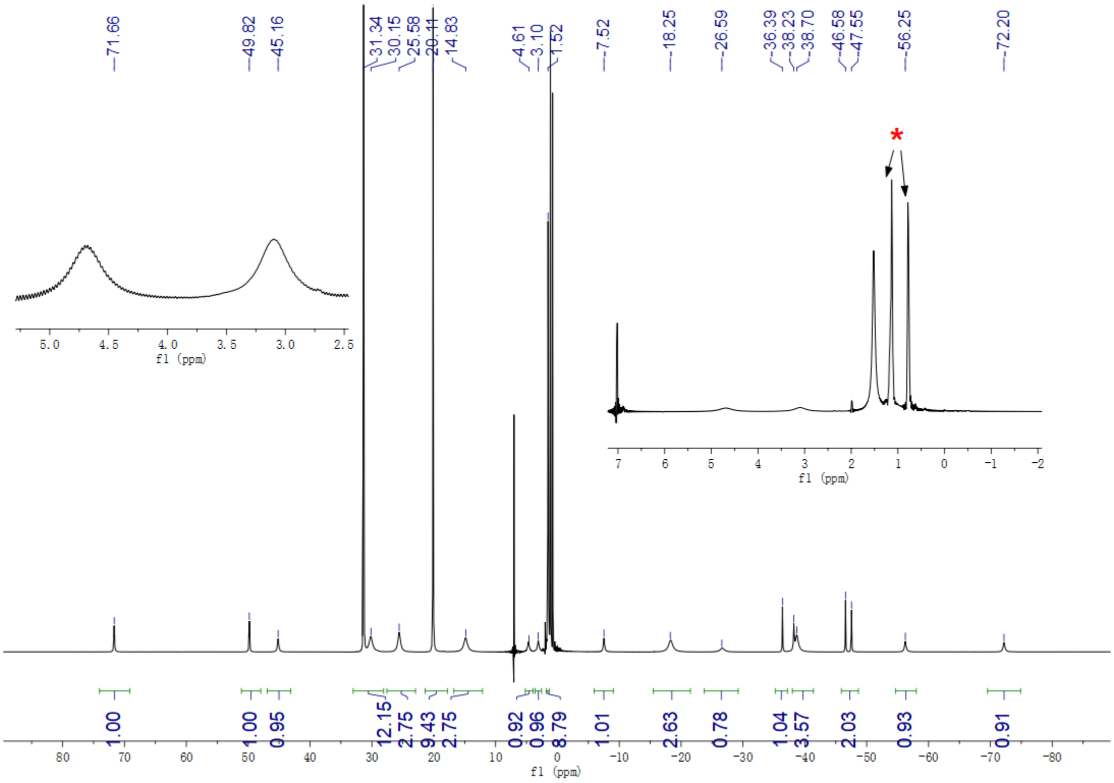

Figure S1. ^{1}H NMR spectra of **1** in C_6D_6 at 298 K. Asterisk are relative to residual *n*-pentane.

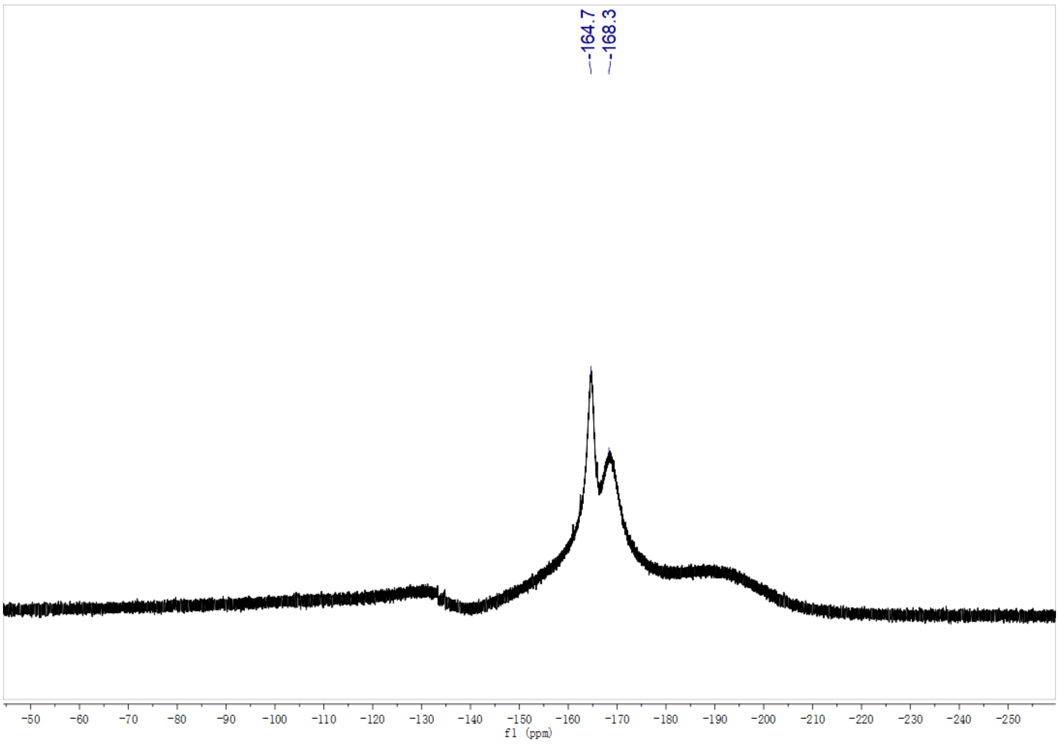

Figure S2. $^{19}F\{^{1}H\}$ NMR spectra of **1** in C_6D_6 at 298 K.

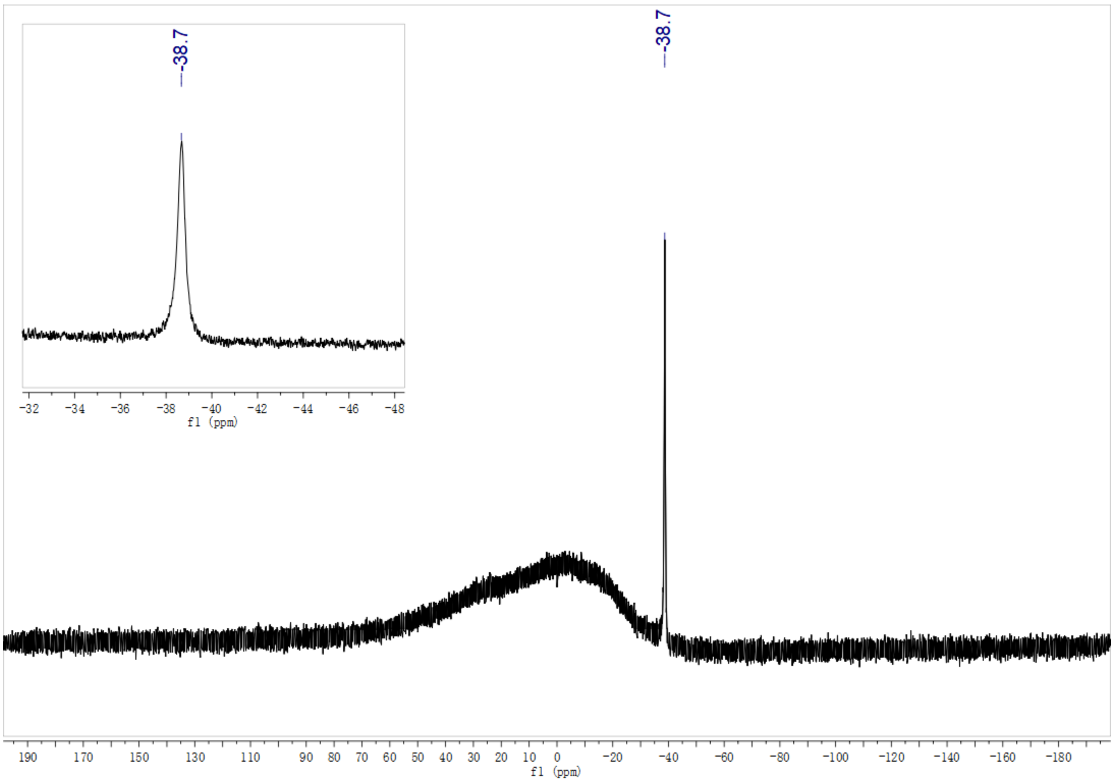

Figure S3. ^{11}B NMR spectra of **1** in C_6D_6 at 298 K.

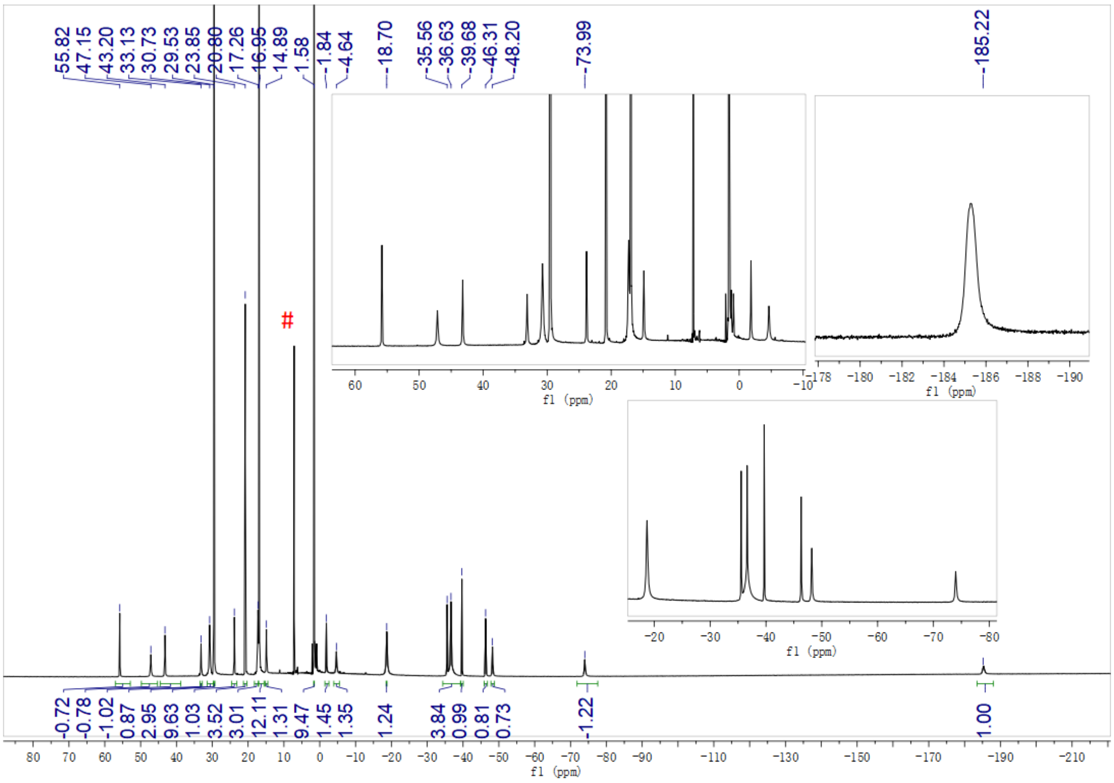

Figure S4. ^{1}H NMR spectra of **2** in C_6D_6 at 298 K. Pound is relative to residual *C_6D_6*

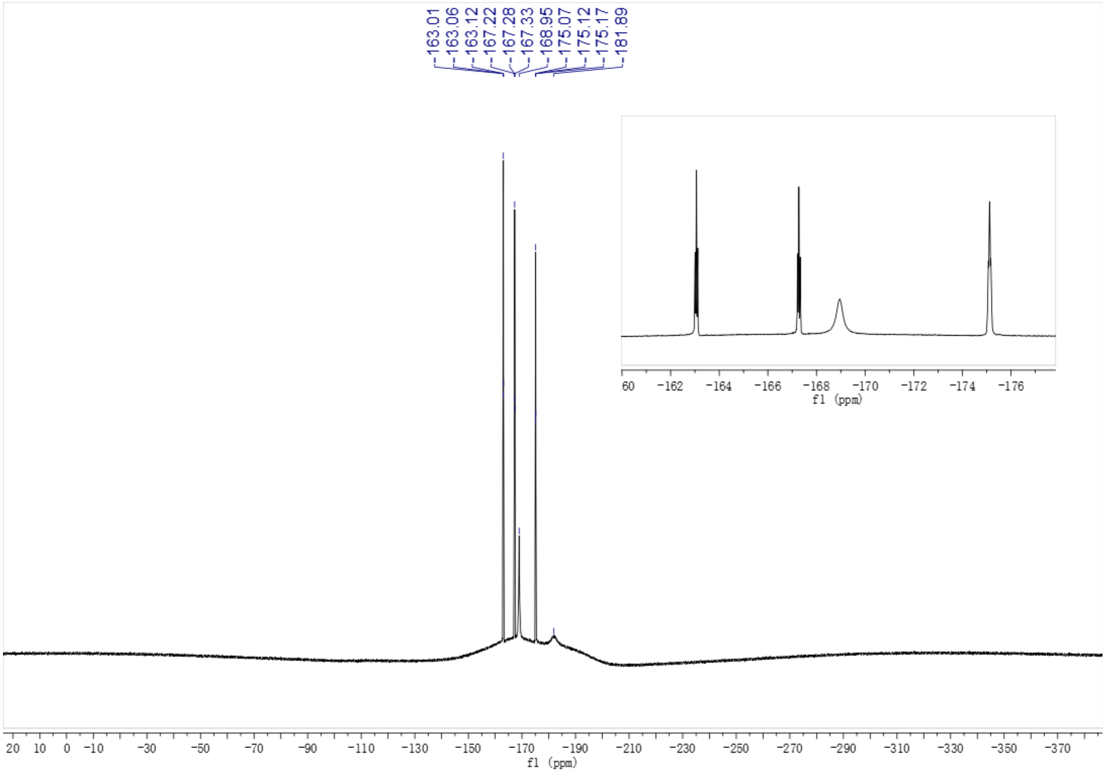

Figure S5. $^{19}F\{^{1}H\}$ NMR spectra of **2** in C_6D_6 at 298 K.

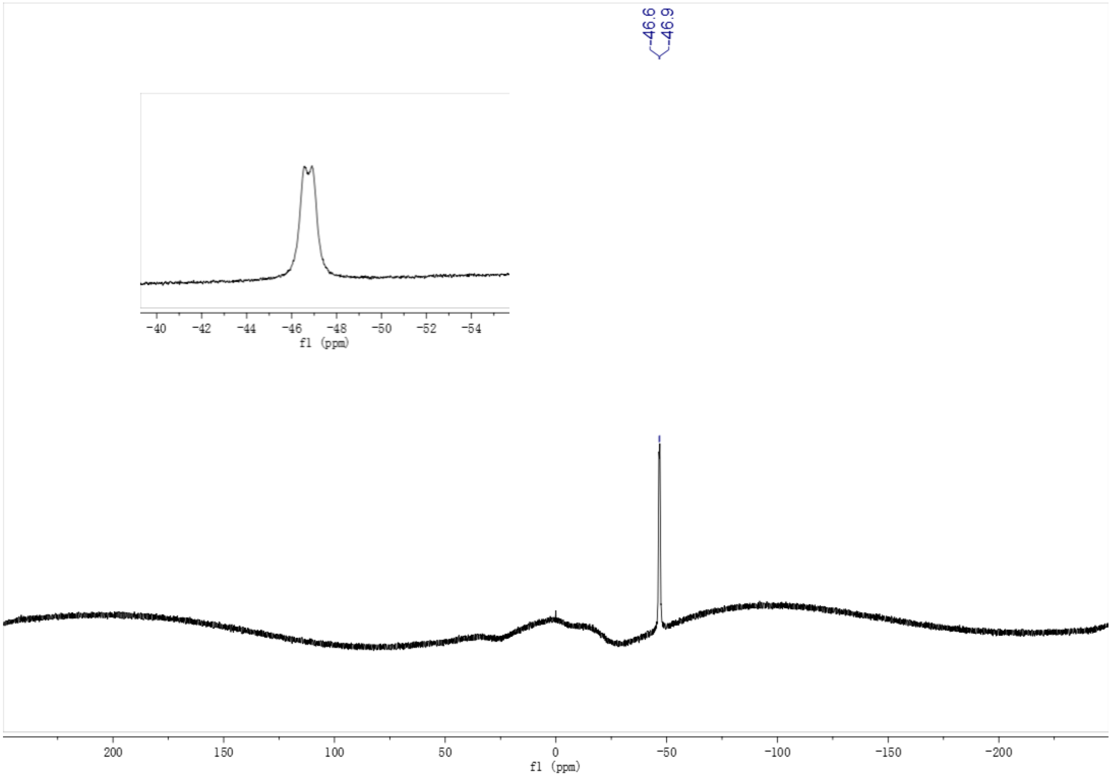

Figure S6. ^{11}B NMR spectra of **2** in C_6D_6 at 298 K.

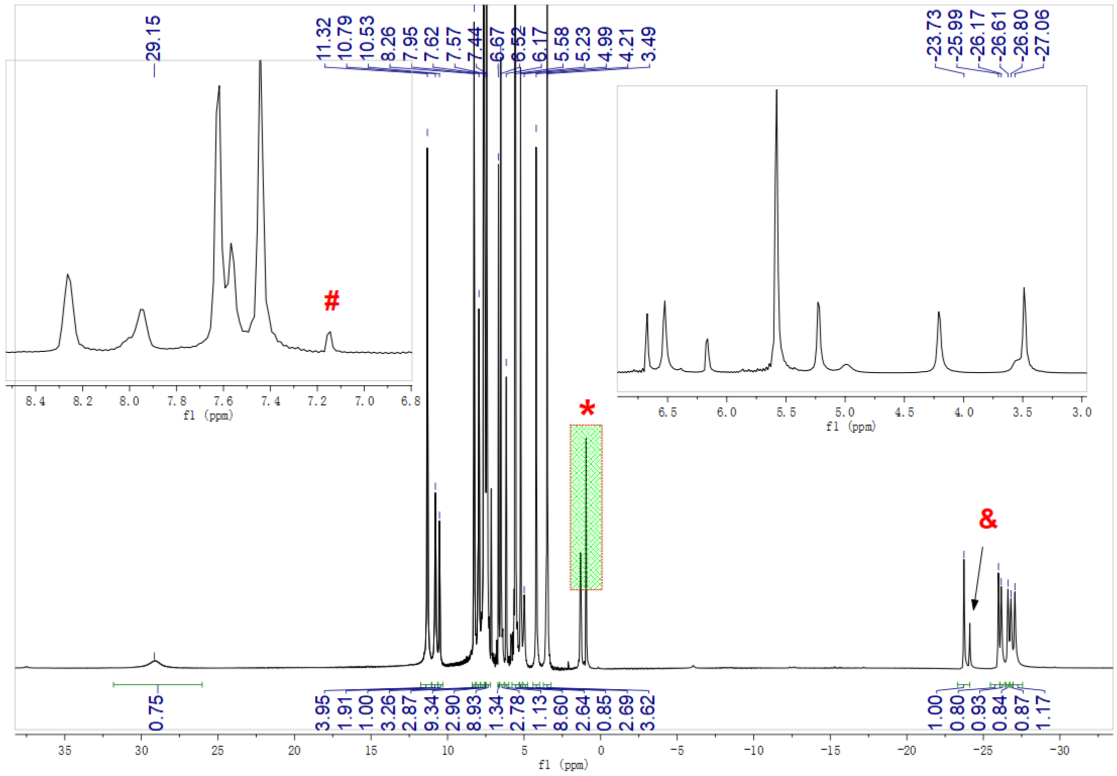

Figure S7. ^{1}H NMR spectra of **3** in C_6D_6 at 298 K. Pound is residual solvent C_6D_6; asterisk is relative to residual *n*-pentane; Ampersand relative to complex **6**.

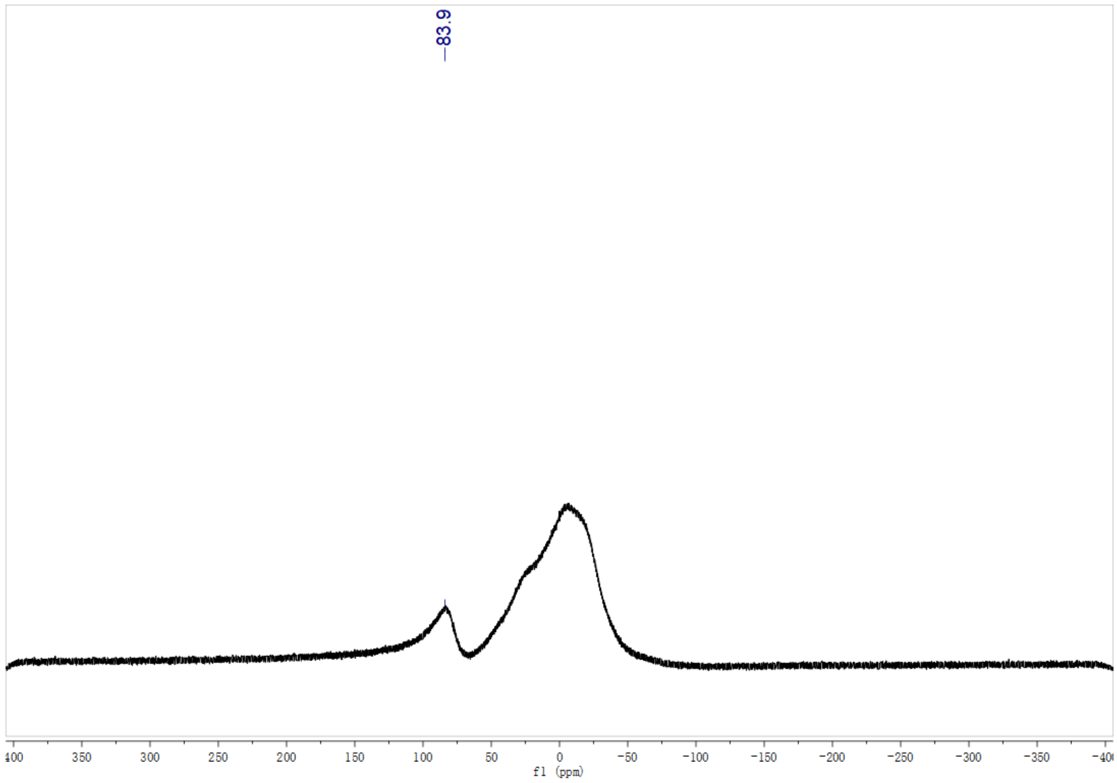

Figure S8. ^{11}B NMR spectra of **3** in C_6D_6 at 298 K.

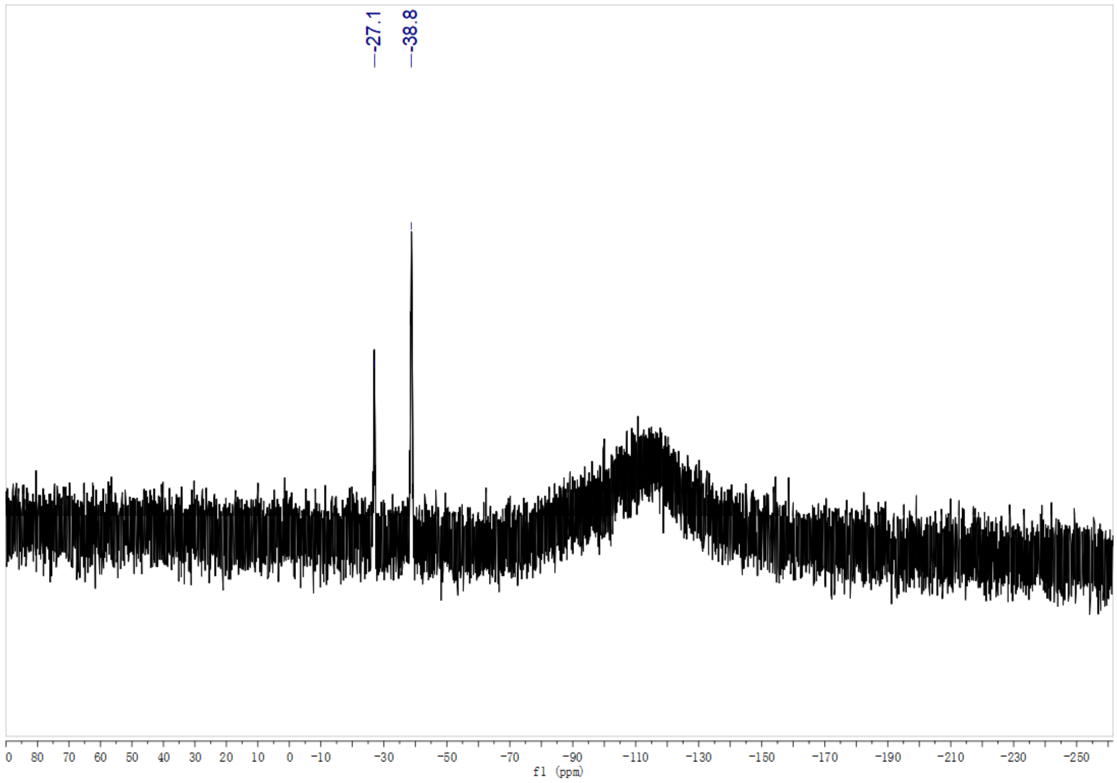

Figure S9. ^{29}Si NMR spectra of **3** in C_6D_6 at 298 K.

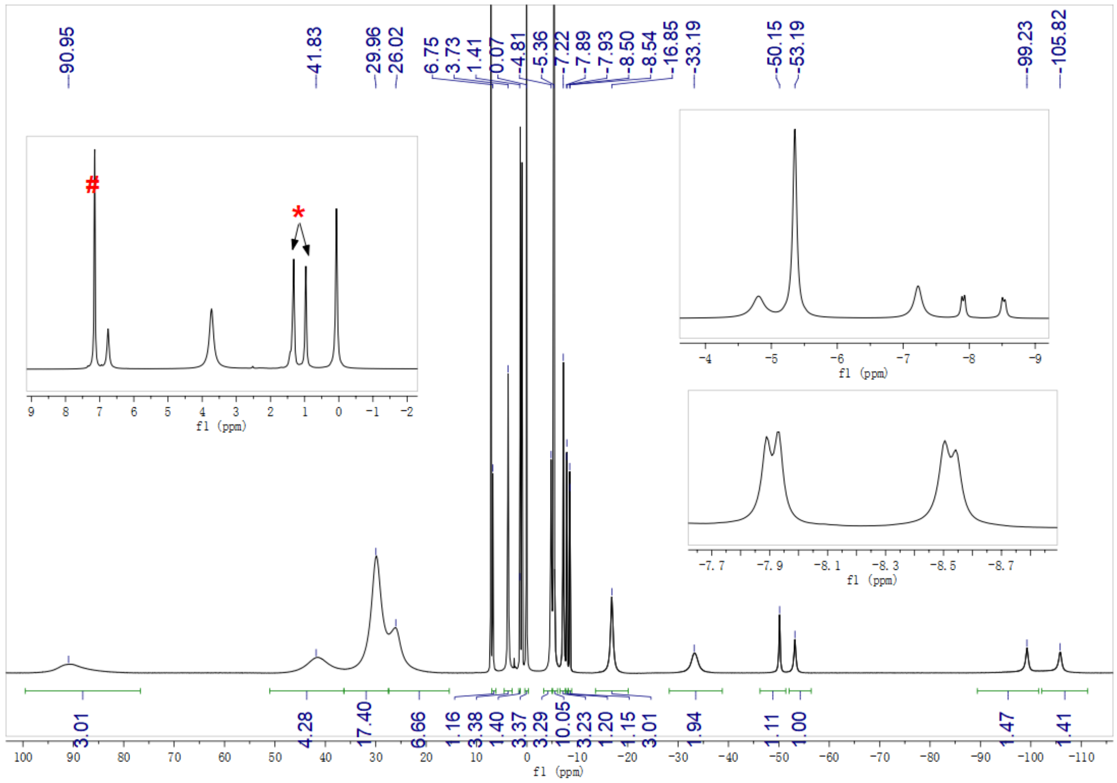

Figure S10. ^1H NMR spectra of **4** in C_6D_6 at 298 K. Pound is residual solvent C_6D_6; asterisk is relative to residual *n*-pentane.

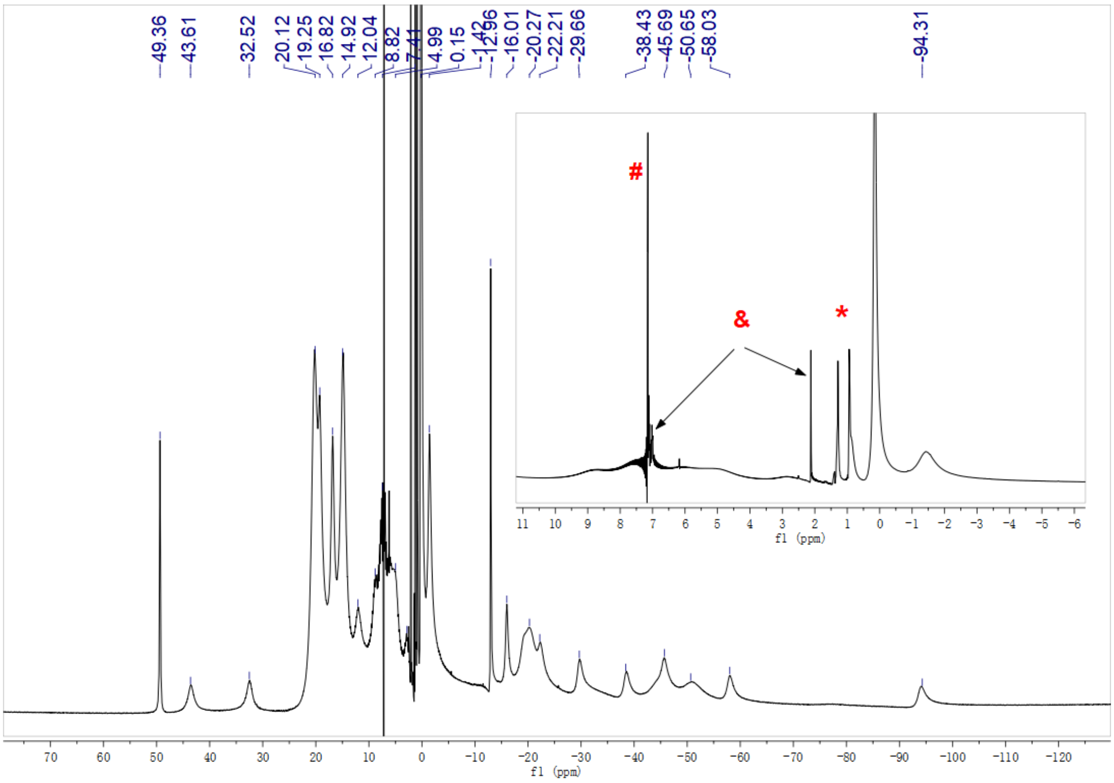

Figure S11. ^{1}H NMR spectra of **5** in C_6D_6 at 298 K. Pound is residual solvent C_6D_6, ampersand is relative to residual toluene, asterisk to n-pentane.

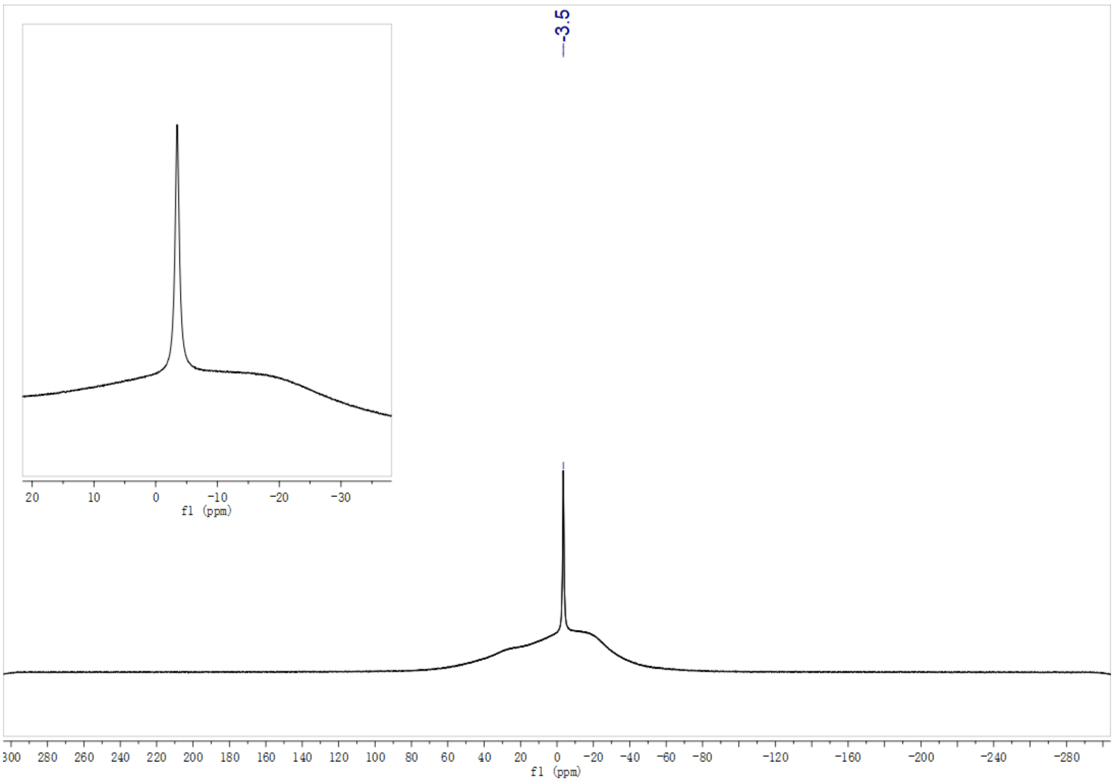

Figure S12. ^{11}B NMR spectra of **5** in C_6D_6 at 298 K.

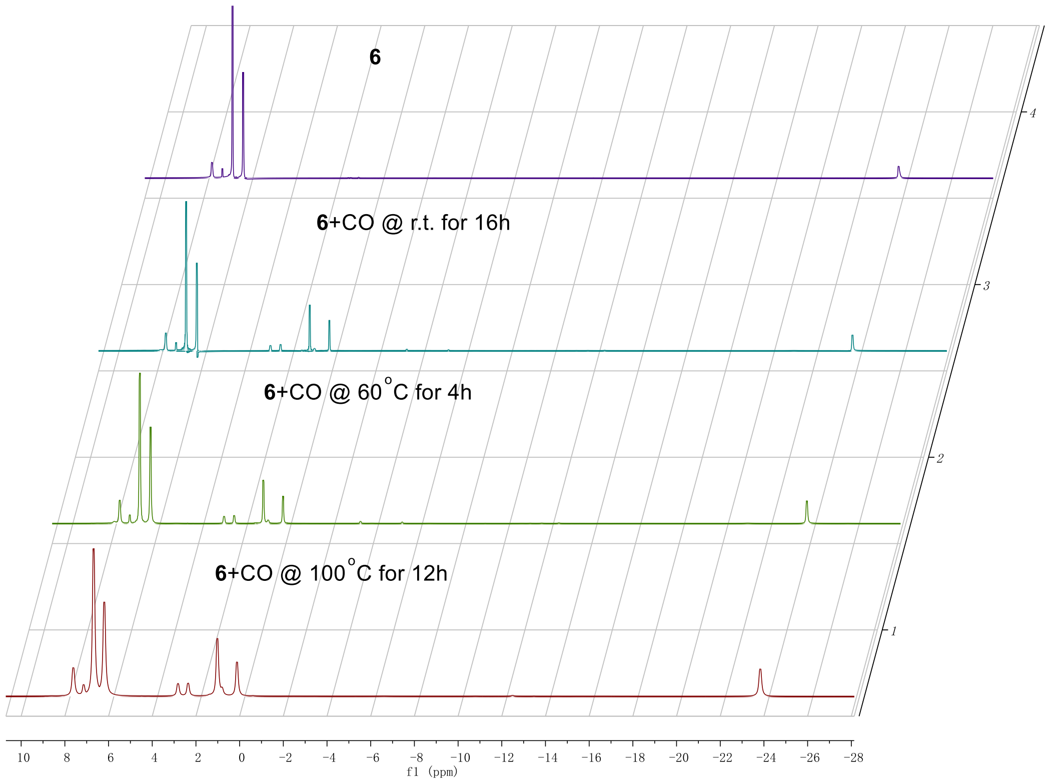

Figure S13. ^{1}H NMR spectral changes of **6** exposed to CO at various temperature and reaction time.

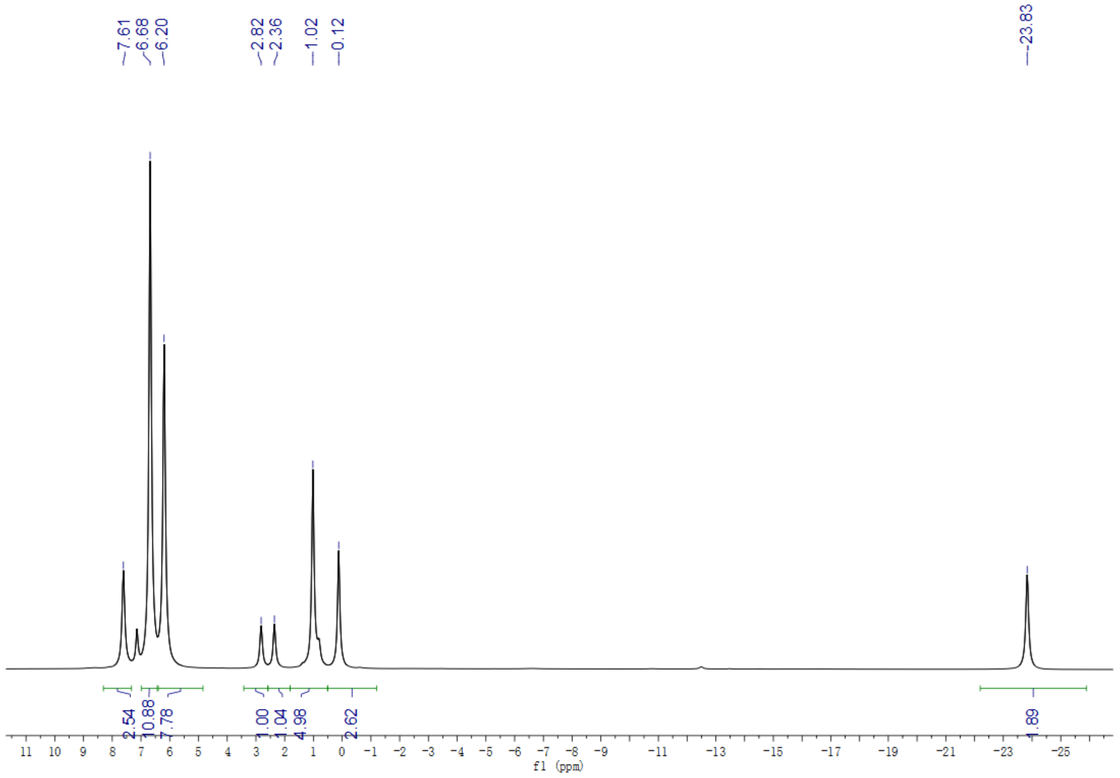

Figure S14. ^{1}H NMR spectra of **6** exposed to CO at 100 °C for 12h.

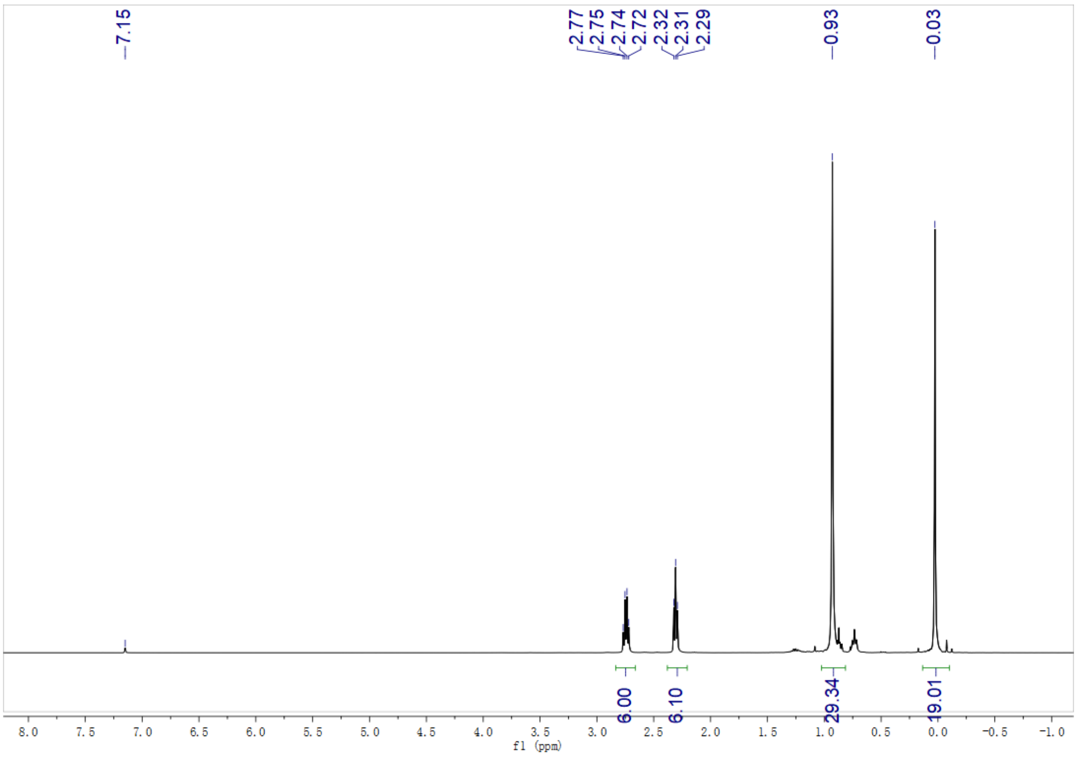

Figure S15. ^1H NMR spectra of the free ligand H_3L.

Magnetic properties

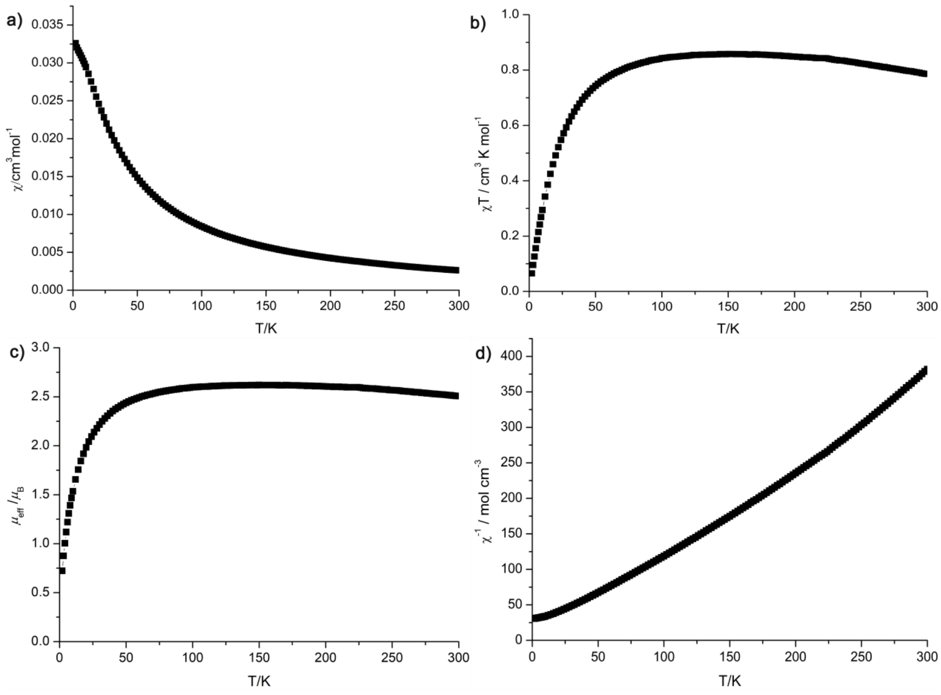

Figure S16. Variable temperature SQUID magnetisation for **1**, presented as: a) χ vs. T; b) χT vs. T; c) μ_{eff} vs. T and d) χ^{-1} vs. T.

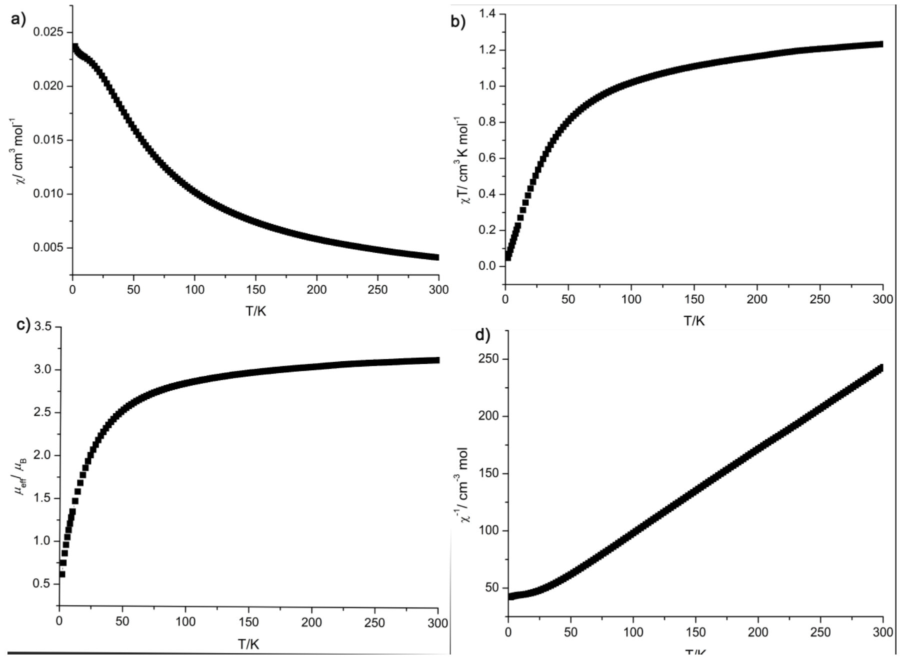

Figure S17. Variable temperature SQUID magnetisation for **2**. presented as: a) χ vs. T; b) χT vs. T; c) μ_{eff} vs. T and d) χ^{-1} vs. T.

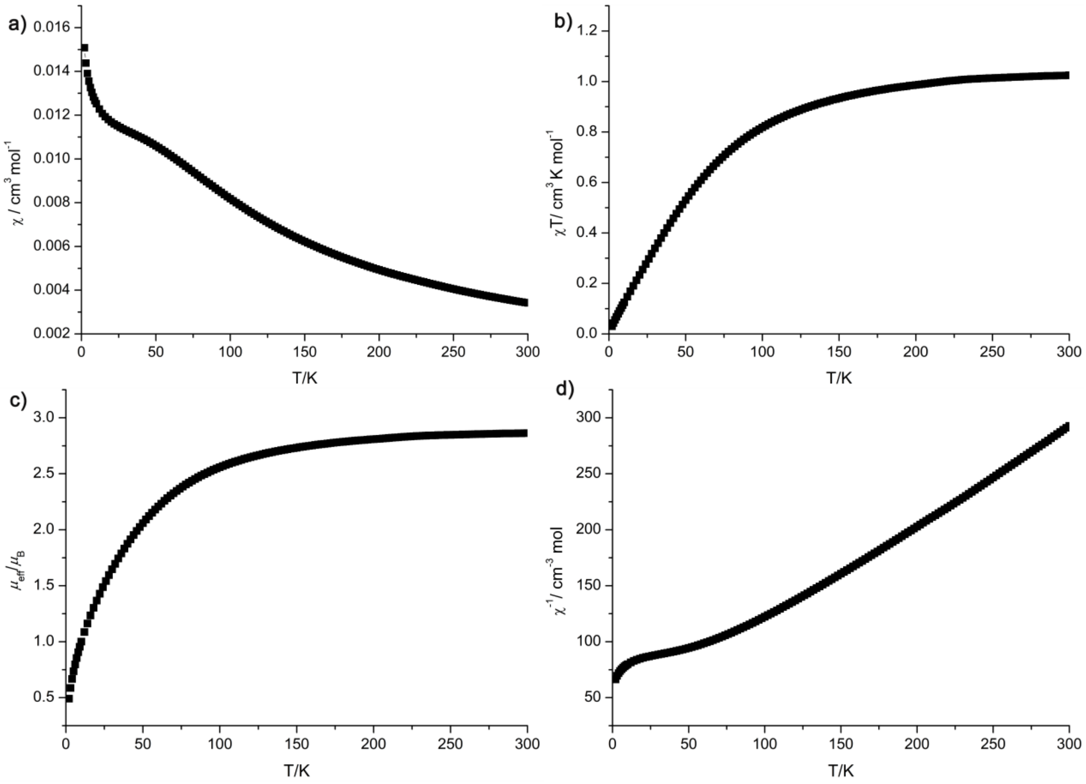

Figure S18. Variable temperature SQUID magnetisation for **3**. presented as: a) χ vs. T; b) χT vs. T; c) μ_{eff} vs. T and d) χ^{-1} vs. T.

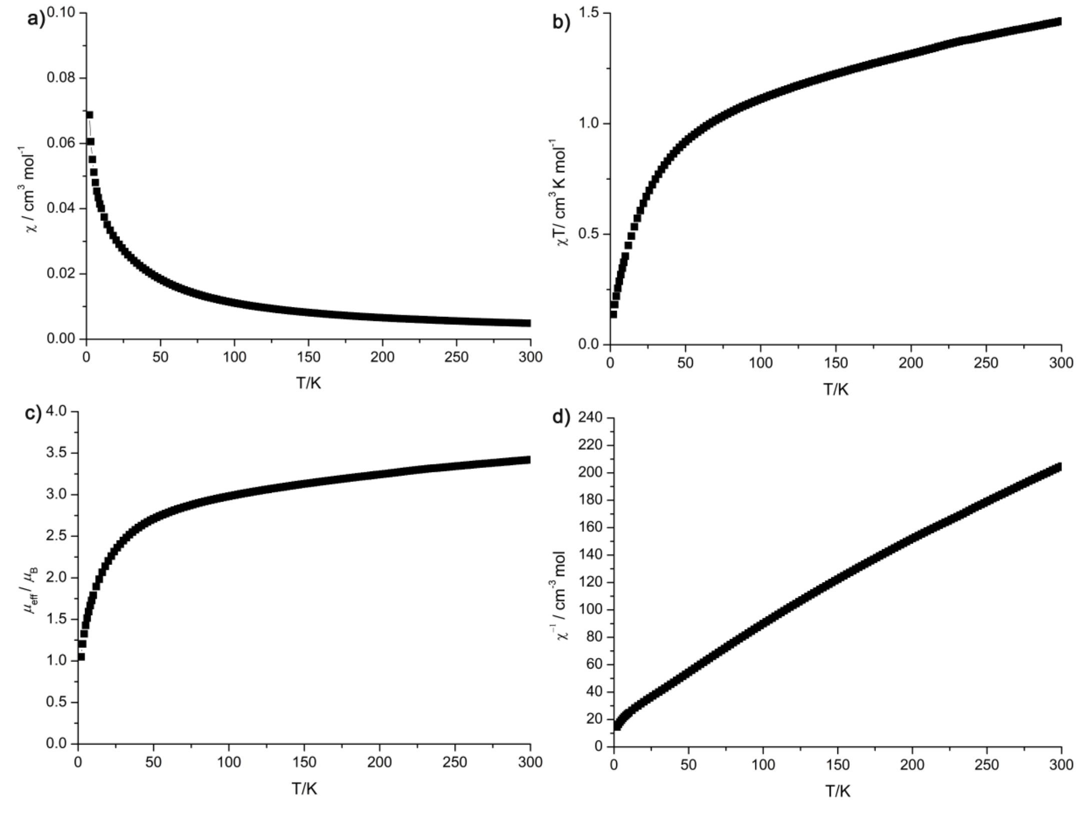

Figure S19. Variable temperature SQUID magnetisation for **4**. presented as: a) χ vs. T; b) χT vs. T; c) μ_{eff} vs. T and d) χ^{-1} vs. T.

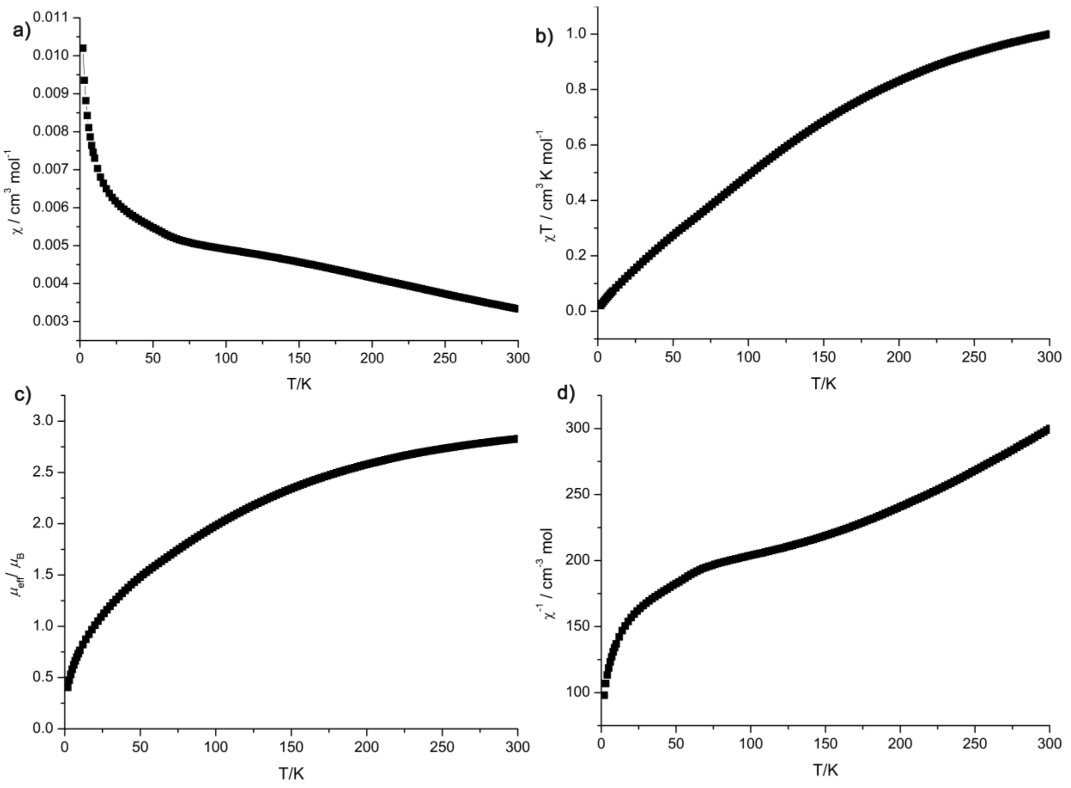

Figure S20. Variable temperature SQUID magnetisation for **5**. presented as: a) χ vs. T; b) χT vs. T; c) μ_{eff} vs. T and d) χ^{-1} vs. T.

Infrared spectroscopy

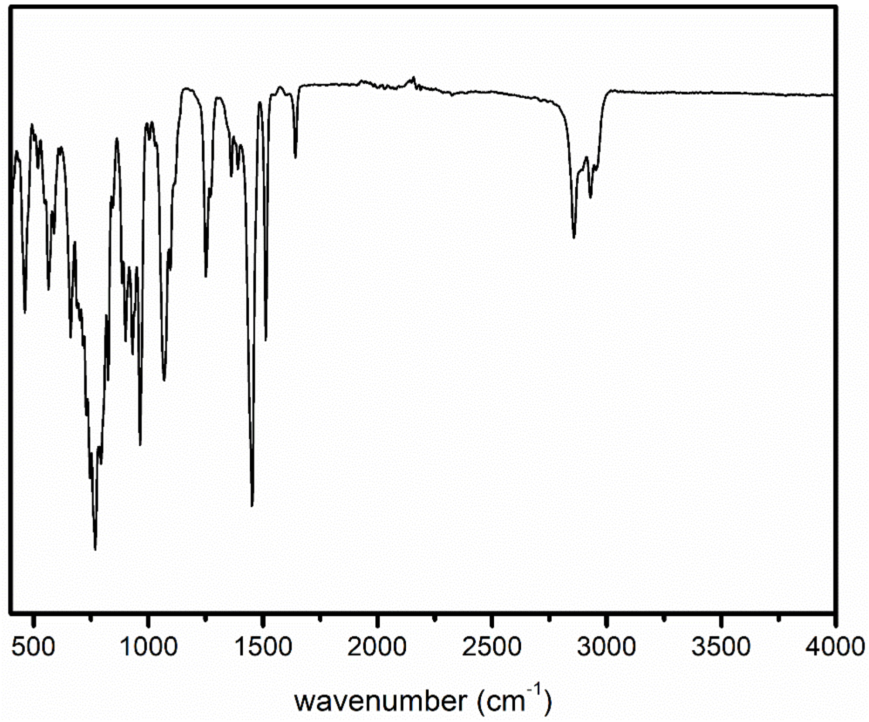

Figure S21. FTIR of **1** in the solid state.

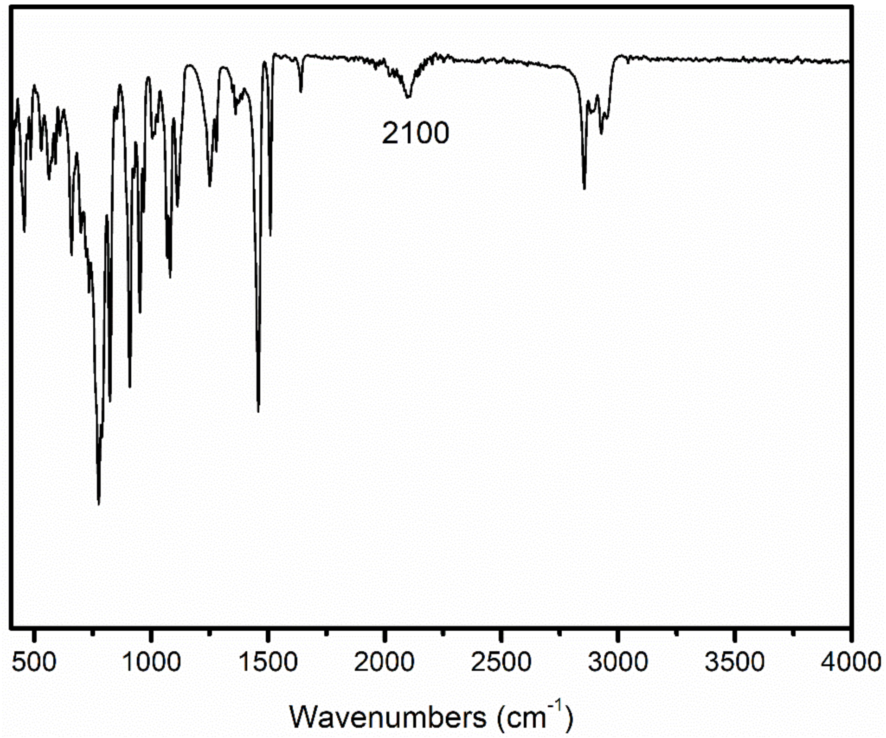

Figure S22. FTIR of **2** in the solid state.

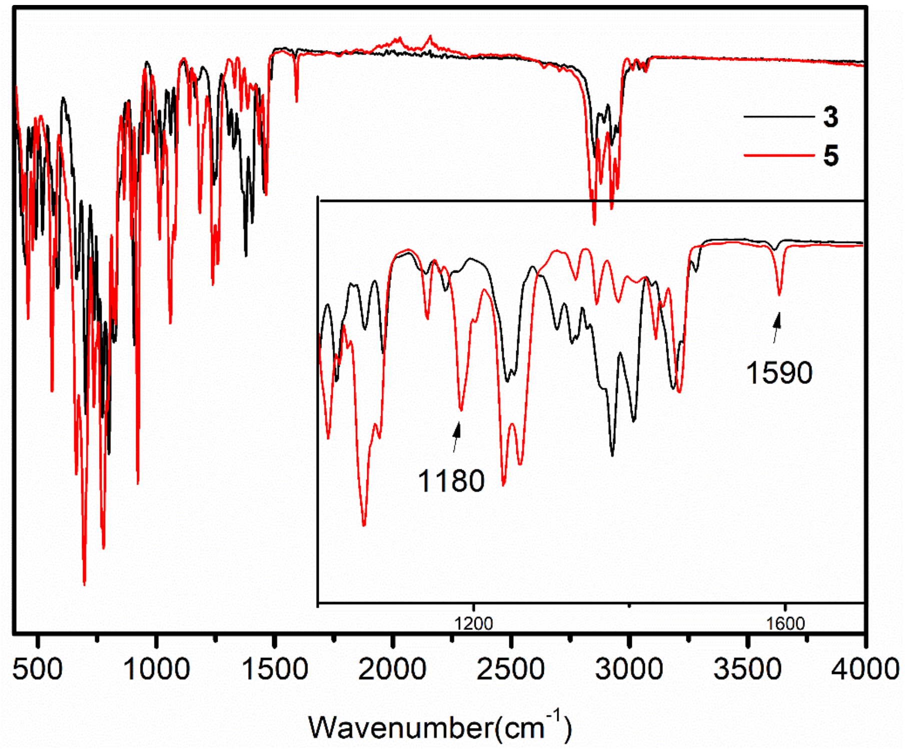

Figure S23. FTIR of **3** and **5** in the solid state.

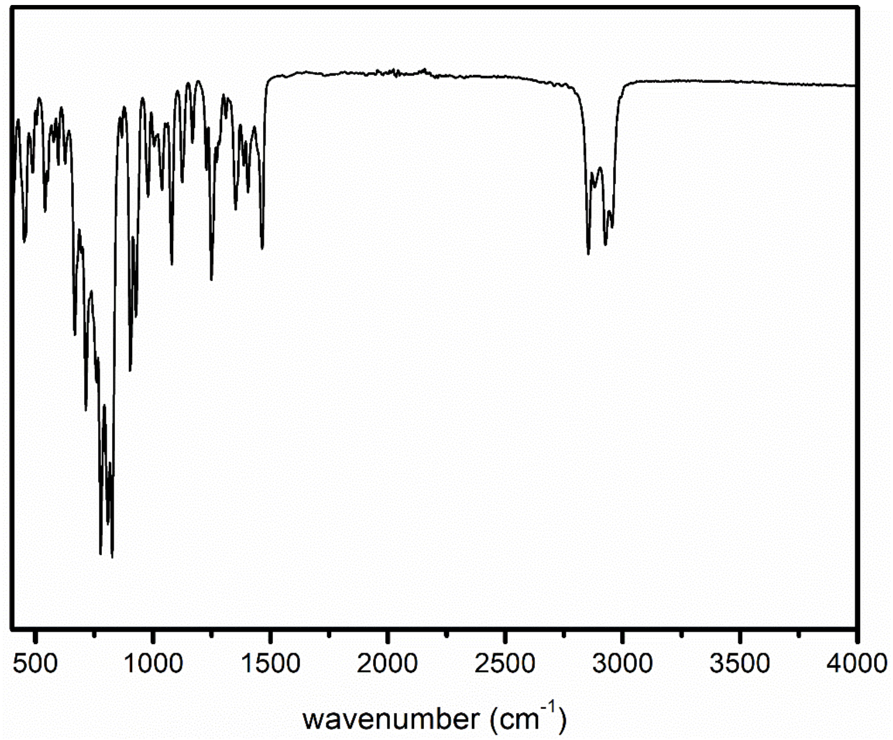

Figure S24. FTIR of **4** in the solid state.

Single-crystal XRD

The intensity data were collected with a Bruker APEX-II CCD area detector using graphite-monochromated Mo Kα radiation (λ = 0.71073 Å). Multiscan or empirical absorption corrections (SADABS) were applied. The structures were solved by Patterson methods, expanded by difference Fourier syntheses, and refined by full-matrix least squares on F^2 using the Bruker SHELXTL-2014 program package[6] using OLEX[7]. All non-hydrogen atoms were refined anisotropically. Hydrogen atoms were introduced at their geometric positions and refined as riding atoms. The X-ray crystal structures have been deposited in the Cambridge Crystallographic Data Centre (CCDC) CODE. CCDC 2204385 (**1**), 2204386 (**2**), 2204387 (**3**), 2204388 (**4**), 2204389 (**5**). The data can be obtained free of charge from the CCDC (www.ccdc.cam.ac.uk/data_request/cif). Details of the data collection and refinement for complexes **1**–**5** are given in Table S1-S5.

**Table S1.** Crystal data and structure refinement for **1•**(1.5C_6H_{14}).	
Empirical formula	$C_{51}H_{77}BF_{15}N_4Si_3U$
Formula weight	1364.27
Temperature/K	100
Crystal system	triclinic
Space group	P-1
a/Å	12.912(10)
b/Å	13.219(11)
c/Å	19.412(19)
α/°	102.48(4)
β/°	103.82(4)
γ/°	105.37(4)
Volume/Å^3	2961(5)
Z	2
ρ_{calc}g/cm^3	1.530
μ/mm^{-1}	2.886
F(000)	1370.0
Crystal size/mm^3	0.08 × 0.06 × 0.06
Radiation	MoKα (λ = 0.71073)
2Θ range for data collection/°	4.486 to 49.998
Index ranges	-15 ≤ h ≤ 15, -15 ≤ k ≤ 15, -23 ≤ l ≤ 23
Reflections collected	106281
Independent reflections	10427 [R_{int}= 0.0849, R_{sigma}= 0.0372]
Data/restraints/parameters	10427/0/729
Goodness-of-fit on F^2	1.051

Final R indexes [I>=2σ (I)]	R_1= 0.0272, wR_2= 0.0680
Final R indexes [all data]	R_1= 0.0294, wR_2= 0.0689
Largest diff. peak/hole / e $Å^{-3}$	1.64/-1.59

Table S2. Crystal data and structure refinement for **2.**

Empirical formula	$C_{36}H_{57}BF_{10}N_4Si_3U$
Formula weight	1068.96
Temperature/K	100
Crystal system	monoclinic
Space group	$P2_1/n$
a/Å	11.8153(7)
b/Å	18.7918(12)
c/Å	19.6337(11)
α/°	90
β/°	96.491(3)
γ/°	90
Volume/Å^3	4331.3(4)
Z	4
ρ_{calc}g/cm^3	1.639
μ/mm^{-1}	3.905
F(000)	2120.0
Crystal size/mm^3	0.02 × 0.02 × 0.02
Radiation	MoKα (λ = 0.71073)
2Θ range for data collection/°	4.412 to 49.998
Index ranges	-14 ≤ h ≤ 14, -22 ≤ k ≤ 22, -23 ≤ l ≤ 23
Reflections collected	110668
Independent reflections	7621 [R_{int}= 0.1086, R_{sigma}= 0.0371]
Data/restraints/parameters	7621/252/591
Goodness-of-fit on F^2	1.040

Final R indexes [I>=2σ (I)]	R_1= 0.0236, wR_2= 0.0481
Final R indexes [all data]	R_1= 0.0352, wR_2= 0.0510
Largest diff. peak/hole / e Å^{-3}	0.51/-0.56

Table S3. Crystal data and structure refinement for **3**.

Empirical formula	$C_{36}H_{66}BClN_4Si_3U$
Formula weight	923.48
Temperature/K	103.08
Crystal system	triclinic
Space group	P-1
a/Å	12.4289(11)
b/Å	13.2845(11)
c/Å	13.7228(10)
α/°	96.465(4)
β/°	107.744(4)
γ/°	94.826(4)
Volume/Å^3	2127.4(3)
Z	2
ρ_{calc}g/cm^3	1.442
μ/mm^{-1}	3.991
F(000)	932.0
Crystal size/mm^3	0.2 × 0.1 × 0.08
Radiation	MoKα (λ = 0.71073)
2Θ range for data collection/°	4.36 to 50
Index ranges	-14 ≤ h ≤ 14, -15 ≤ k ≤ 15, -16 ≤ l ≤ 16
Reflections collected	59025
Independent reflections	7488 [R_{int}= 0.0888, R_{sigma}= 0.0458]
Data/restraints/parameters	7488/0/429
Goodness-of-fit on F^2	1.034

Final R indexes [I>=2σ (I)]	R_1= 0.0236, wR_2= 0.0542
Final R indexes [all data]	R_1= 0.0289, wR_2= 0.0555
Largest diff. peak/hole / e Å^{-3}	0.83/-0.78

Table S4. Crystal data and structure refinement for 0.5•**4.**

Empirical formula	$C_{34}H_{69}BCl_2N_4Si_3U$
Formula weight	937.94
Temperature/K	100
Crystal system	monoclinic
Space group	$P2_1/n$
a/Å	15.688(9)
b/Å	13.484(5)
c/Å	22.194(10)
α/°	90
β/°	95.86(2)
γ/°	90
Volume/Å^3	4670(4)
Z	4
ρ_{calc}g/cm^3	1.334
μ/mm^{-1}	3.692
F(000)	1896.0
Crystal size/mm^3	0.02 × 0.02 × 0.02
Radiation	MoKα (λ = 0.71073)
2Θ range for data collection/°	4.508 to 49.996
Index ranges	-18 ≤ h ≤ 18, -16 ≤ k ≤ 16, -26 ≤ l ≤ 26
Reflections collected	64012
Independent reflections	8223 [R_{int}= 0.1128, R_{sigma}= 0.0604]
Data/restraints/parameters	8223/234/483

Goodness-of-fit on F^2	1.019
Final R indexes [I>=2σ (I)]	R_1= 0.0350, wR_2= 0.0738
Final R indexes [all data]	R_1= 0.0595, wR_2= 0.0801
Largest diff. peak/hole / e $Å^{-3}$	0.98/-0.85

Table S5. Crystal data and structure refinement for **5**.

Empirical formula	$C_{41}H_{74}BClN_4O_2Si_3U$
Formula weight	1023.60
Temperature/K	100.02
Crystal system	monoclinic
Space group	$P2_1/n$
a/Å	17.1714(15)
b/Å	11.7801(10)
c/Å	29.615(3)
α/°	90
β/°	102.509(3)
γ/°	90
Volume/Å^3	5848.3(9)
Z	4
ρ_{calc}g/cm^3	1.163
μ/mm^{-1}	2.912
F(000)	2080.0
Crystal size/mm^3	0.1 × 0.1 × 0.08
Radiation	MoKα (λ = 0.71073)
2Θ range for data collection/°	4.618 to 50
Index ranges	$-20 \leq h \leq 20$, $-14 \leq k \leq 14$, $-35 \leq l \leq 35$
Reflections collected	83502
Independent reflections	10281 [R_{int}= 0.0753, R_{sigma}= 0.0421]

Data/restraints/parameters	10281/987/570
Goodness-of-fit on F^2	1.065
Final R indexes [I>=2σ (I)]	R_1= 0.0467, wR_2= 0.1089
Final R indexes [all data]	R_1= 0.0654, wR_2= 0.1144
Largest diff. peak/hole / e $Å^{-3}$	2.01/-1.49

Computational details

The optimization of different spin states for uranium complexes was carried out by employing DFT hybrid functional (B3PW91)[8] along with Stuttgart small core pseudopotential and associated basis set for uranium, silicon and chlorine atoms with additional polarization functions for silicon and chlorine atoms.[9] Pople basis sets (6-31G**) were employed for the rest of the atoms.[10] Frequency calculations were performed to locate minima (maxima for transition state structures) for the optimized structures. To account for the solvation effects, SMD model using THF solvent has been included in the calculations[11] All the calculations were performed using Gaussian 09 suite of programs.[12]

Table S6: DFT Computed Natural charges for TS1 (s=1)

Atom Label	Natural charges
U1	1.73340
N6	-1.27175
N7	-1.26512
N8	-0.55624
N24	-1.26654
B37	0.69928
O5	-0.43372
C9	0.64363

Table S7 Computed Wiberg bond index for TS1 (s=1)

Atom Label	Wiberg bond index	Atom Label	Wiberg bond index	Atom Label	Wiberg bond index	Atom Label	Wiberg bond index
U1	**0.0000**	U1	0.0302	U1	0.0043	U1	0.0092
N6	0.7511	N6	0.0047	N6	0.0061	N6	0.0021
N7	0.7838	N7	0.0002	N7	0.0000	N7	0.0000
N8	0.2717	N8	0.0000	N8	0.0000	N8	0.0000
N24	0.7623	N24	0.0003	N24	0.0001	N24	0.0001
B37	0.0043	B37	0.3651	B37	**0.0000**	B37	0.0054
O5	0.0092	O5	2.2699	O5	0.0054	O5	**0.0000**
C9	0.0302	C9	**0.0000**	C9	0.3651	C9	2.2699

Table S8: DFT Computed Natural charges for TS2 (s=1)

Atom Label	Natural charges
U1	1.75494
N6	-1.23212
N7	-1.27094
N8	-0.56807
N24	-1.27132
B37	0.27479
O5	-0.40148
C9	0.72670

Table S9: Computed Wiberg bond index for TS2 (s=1)

Atom Label	Wiberg bond index	Atom Label	Wiberg bond index	Atom Label	Wiberg bond index	Atom Label	Wiberg bond index
U1	**0.0000**	U1	0.0429	U1	0.0043	U1	0.0543
N6	0.5537	N6	0.2268	N6	0.0061	N6	0.0756
N7	0.7845	N7	0.0004	N7	0.0000	N7	0.0008
N8	0.2644	N8	0.0003	N8	0.0000	N8	0.0001
N24	0.7864	N24	0.0011	N24	0.0001	N24	0.0004
B37	0.0124	B37	0.8785	B37	**0.0000**	B37	0.0150
O5	0.0543	O5	2.1359	O5	0.0054	O5	**0.0000**
C9	0.0302	C9	**0.0000**	C9	0.3651	C9	2.1359

Table S10: Natural Bond Analysis for TS2

(0.98117) BD (1) C 9 - B 37

(68.58%) 0.8281* C 9 s(67.95%)p 0.47(32.04%)d 0.00(0.01%)

(31.42%) 0.5605* B 37 s(19.07%)p 4.24(80.80%)d 0.01(0.13%)

(0.99830) BD (1) O 5 - C 9

(69.32%) 0.8326* O 5 s(49.04%)p 1.03(50.55%)d 0.01(0.41%)

(30.68%) 0.5539* C 9 s(30.14%)p 2.31(69.67%)d 0.01(0.20%)

(0.99678) BD (2) O 5 - C 9

(74.01%) 0.8603* O 5 s(0.00%)p 1.00(99.59%)d 0.00(0.41%)

(25.99%) 0.5098* C 9 s(0.36%)p99.99(99.32%)d 0.92(0.33%)

(0.99301) BD (3) O 5 - C 9

(75.02%) 0.8661* O 5 s(0.12%)p99.99(99.50%)d 3.31(0.38%)

(24.98%) 0.4998* C 9 s(2.08%)p47.00(97.59%)d 0.16(0.34%)

Table S11: DFT computed NBO second order perturbation analysis for TS2

Donor NBO	Acceptor NBO	E(2) kcal/mol
7. (0.99830) BD (1) O 5 - C 9<br>(69.32%) 0.8326* O 5 s(49.04%)p 1.03(50.55%)d 0.01(0.41%)<br>(30.68%) 0.5539* C 9 s(30.14%)p 2.31(69.67%)d 0.01(0.20%)	179. (0.15696) LP*(5) U 1<br>s(0.43%)p3.32(1.43%)d99.99(76.16%)f50.98(21.97%) g 0.01(0.00%)	12.77
9. (0.99301) BD (3) O 5 - C 9<br>(75.02%) 0.8661* O 5 s(0.12%)p99.99(99.50%)d 3.31(0.38%)<br>(24.98%) 0.4998* C 9 s(2.08%)p47.00(97.59%)d 0.16(0.34%)	179. (0.15696) LP*(5) U 1<br>s(0.43%)p3.32(1.43%)d99.99(76.16%)f50.98(21.97%) g 0.01(0.00%)	150.76
198. (0.88799) LP (1) N 6<br>s(9.01%)p10.10(90.99%)d 0.00(0.01%)	366. (0.00076) RY*(4) C 9<br>s(0.44%)p62.79(27.68%)d99.99(71.88%)	165.04
199. (0.82091) LP (2) N 6<br>s(53.80%)p 0.86(46.15%)d 0.00(0.05%)	366. (0.00076) RY*(4) C 9<br>s(0.44%)p62.79(27.68%)d99.99(71.88%)	41.23
200. (0.80098) LP (3) N 6<br>s(0.02%)p99.99(99.97%)d 0.69(0.01%)	366. (0.00076) RY*(4) C 9<br>s(0.44%)p62.79(27.68%)d99.99(71.88%)	161.59

Table S12: DFT Computed Natural charges for **5** (s=1)

**Atom Label**	**Natural charges**
U1	1.48715
N6	-0.80748
N8	-1.24199
N9	-0.53942
N25	-1.22680
B41	0.33928
O5	-0.69937
C10	0.57664
O7(THF)	-0.59765

Table S13: Computed Wiberg bond index for **5** (s=1)

**Atom Label**	**Wiberg bond index**	**Atom Label**	**Wiberg bond index**	**Atom Label**	**Wiberg bond index**	**Atom Label**	**Wiberg bond index**
U1	**0.0000**	U1	0.0348	U1	0.0168	U1	0.5793
N6	0.0264	N6	1.3660	N6	0.0101	N6	0.1056
N8	0.8242	N8	0.0025	N8	0.0001	N8	0.0039
N9	0.2794	N9	0.0009	N9	0.0001	N9	0.0015
N25	0.8691	N25	0.0025	N25	0.0039	N25	0.0039
B41	0.0168	B41	0.8415	B41	**0.0000**	B41	0.0003
O5	0.5793	O5	1.2517	O5	0.0165	O5	**0.0000**
C10	0.0348	C10	**0.0000**	C10	0.8415	C10	1.2517

Table S14: Natural Bond Analysis for **5**

<u>(0.99364) BD (1) O 5 - C 10</u>

(68.39%) 0.8270* O 5 s(46.90%)p 1.13(53.05%)d 0.00(0.05%)

(31.61%) 0.5622* C 10 s(25.31%)p 2.94(74.43%)d 0.01(0.27%)

<u>(0.98933) BD (1) N 6 - C 10</u>

(63.24%) 0.7952* N 6 s(42.29%)p 1.36(57.67%)d 0.00(0.04%)

(36.76%) 0.6063* C 10 s(28.04%)p 2.56(71.81%)d 0.01(0.15%)

Table S15: DFT computed NBO second order perturbation analysis for **5**

Donor NBO	Acceptor NBO	E(2) kcal/mol
7. (0.99364) BD (1) O 5 - C 10<br>(68.39%) 0.8270* O 5 s(46.90%)p 1.13(53.05%)d 0.00(0.05%)<br>(31.61%) 0.5622* C 10 s(25.31%)p 2.94(74.43%)d 0.01(0.27%)	200. (0.07899) LP*(9) U 1<br>s(18.57%)p 0.24(4.39%)d 2.27(42.13%) f 1.88(34.91%)g 0.00(0.01%)	36.82
7. (0.99364) BD (1) O 5 - C 10<br>(68.39%) 0.8270* O 5 s(46.90%)p 1.13(53.05%)d 0.00(0.05%)<br>(31.61%) 0.5622* C 10 s(25.31%)p 2.94(74.43%)d 0.01(0.27%)	205. (0.03337) LP*(14) U 1<br>s(0.56%)p32.10(17.96%)d79.35(44.39%) f66.29(37.08%)g 0.01(0.01%)	26.53
214. (0.92462) LP (1) O 5<br>s(46.10%)p 1.17(53.88%)d 0.00(0.01%)	194. (0.20928) LP*(3) U 1<br>s(0.82%)p0.06(0.05%)d39.74(32.68%)f80.79(66.44%) g 0.00(0.00%)	12.39
214. (0.92462) LP (1) O 5<br>s(46.10%)p 1.17(53.88%)d 0.00(0.01%)	196. (0.15993) LP*(5) U 1<br>s(0.37%)p 4.02(1.49%)d99.99(83.13%) f40.59(15.01%)g 0.03(0.01%)	37.15
214. (0.92462) LP (1) O 5<br>s(46.10%)p 1.17(53.88%)d 0.00(0.01%)	198. (0.12451) LP*(7) U 1<br>s(5.39%)p 0.38(2.05%)d14.54(78.39%) f 2.63(14.15%)g 0.00(0.01%)	18.69
214. (0.92462) LP (1) O 5<br>s(46.10%)p 1.17(53.88%)d 0.00(0.01%)	202. (0.06096) LP*(11) U 1<br>s(0.23%)p99.99(61.06%)d63.69(14.45%) f99.99(24.26%)g 0.04(0.01%)	40.50

Figure S25: DFT computed unpaired spin density plots (a) TS1 (b) TS2 (c) **5** for s=1 spin state

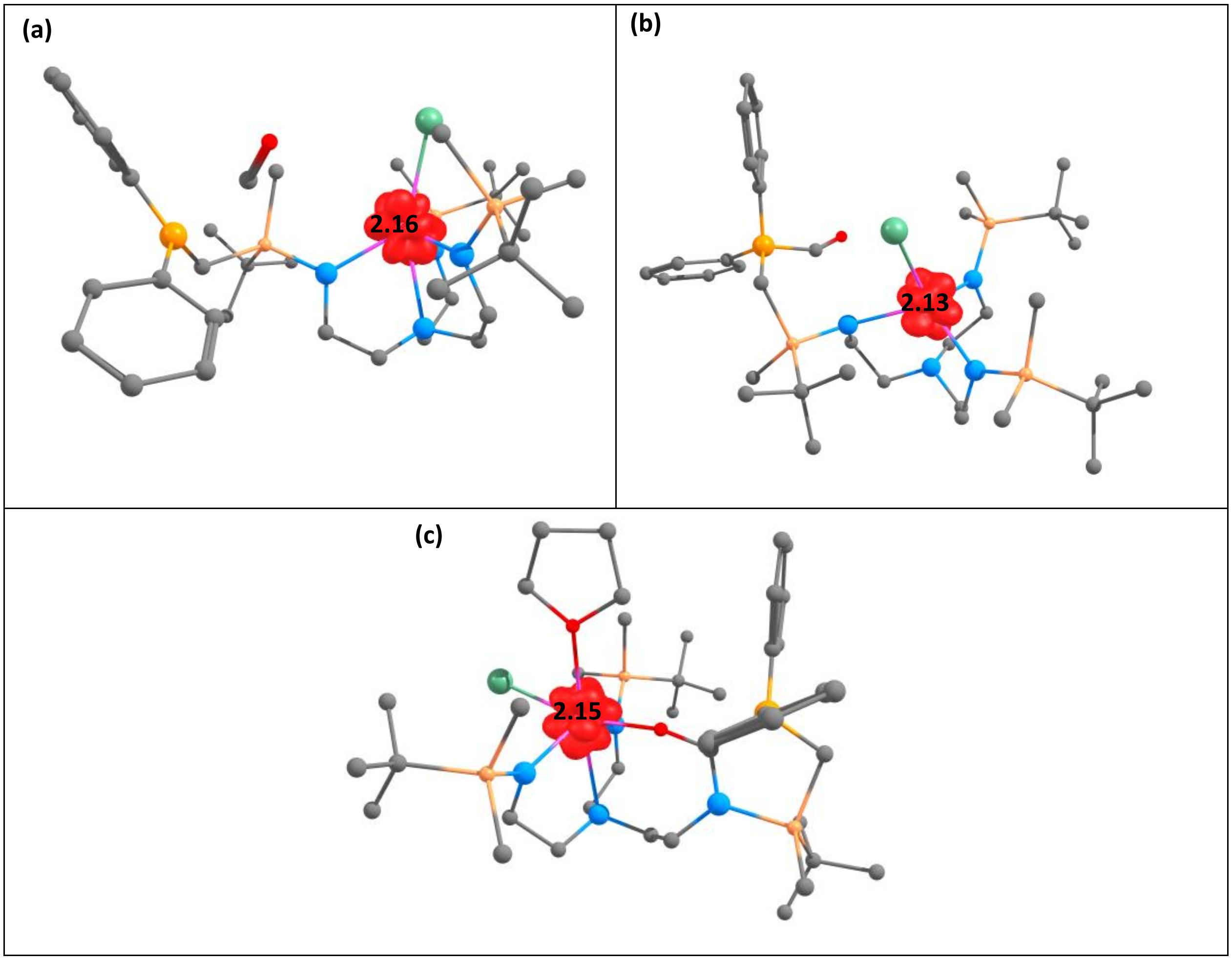

Table S16. Experimental and computed B-H vibration for 2

	**Exp.**	**Calced.**
Frequency (cm^{-1})	2100	2230.5

Table S17. DFT Computed Natural charges for 2 (s=1)

**Atom Label**	**Natural charges**
U1	1.96209
N10	-1.26320
N13	-0.56057
N14	-1.28355
N16	-1.27648
B64	0.19602
H88	-0.07788

Table S18. Computed Wiberg bond index for 2 (s=1)

**Atom Label**	**Wiberg bond index**	**Atom Label**	**Wiberg bond index**
U1	**0.0000**	U1	0.0951
N10	0.8270	N10	0.0033
N13	0.2642	N13	0.0027
N14	0.7677	N14	0.0012
N16	0.7552	N16	0.0039
B64	0.0951	B64	**0.0000**
H88	0.1170	H88	0.8282

Table S19. DFT computed NBO second order perturbation analysis for 2

Donor NBO	Acceptor NBO	E(2) kcal/mol
(0.92937) BD (1) B 64 - H 88<br>(42.67%) 0.6532* B 64 s(22.86%)p 3.37(77.07%)d 0.00(0.07%)<br>(57.33%) 0.7572* H 88 s(99.98%)p 0.00(0.02%)	(0.10189) LP*(7) U 1<br>s(0.24%)p14.41(3.51%)d99.99(69.05%)f99.99(27.18%)g 0.09(0.02%)	7.69
(0.92937) BD (1) B 64 - H 88<br>(42.67%) 0.6532* B 64 s(22.86%)p 3.37(77.07%)d 0.00(0.07%)<br>(57.33%) 0.7572* H 88 s(99.98%)p 0.00(0.02%)	(0.07102) LP*(9) U 1<br>s(56.06%)p 0.08(4.67%)d 0.38(21.14%)f 0.32(18.12%)g 0.00(0.01%)	5.23
(0.92937) BD (1) B 64 - H 88<br>(42.67%) 0.6532* B 64 s(22.86%)p 3.37(77.07%)d 0.00(0.07%)<br>(57.33%) 0.7572* H 88 s(99.98%)p 0.00(0.02%)	(0.04264) LP*(10) U 1<br>s(0.53%)p99.99(92.01%)d 5.08(2.68%)f 9.04(4.77%)g 0.03(0.02%)	4.20

Optimized geometries

2

U	5.671975000	4.480264000	14.508039000
Si	8.241370000	5.134274000	12.257770000
Si	2.667453000	2.722176000	15.311537000
F	5.732995000	6.201370000	8.501397000
F	2.526970000	4.841331000	11.223192000
F	5.863164000	4.494836000	6.443612000
F	0.442855000	6.437268000	10.750539000
F	5.450230000	8.553104000	10.687948000
F	5.642440000	1.805361000	6.863002000
N	7.733358000	4.214721000	13.712756000
F	5.188804000	2.539253000	11.472196000
F	0.825948000	9.097776000	10.241584000
N	7.086872000	3.001361000	16.086526000
N	4.443311000	2.738839000	15.209924000
F	3.361106000	10.107985000	10.221083000
N	6.034965000	5.597018000	16.443286000
F	5.298872000	0.859449000	9.401837000
C	9.754045000	6.315173000	12.619136000
C	8.103004000	2.342751000	15.239936000
H	7.587898000	1.582076000	14.644215000
H	8.852677000	1.828042000	15.861220000
C	5.420976000	4.463059000	10.088321000
C	8.753338000	3.350671000	14.304653000
H	9.504975000	3.937152000	14.855242000
H	9.315768000	2.782355000	13.547815000
C	6.773367000	6.191877000	11.695211000
C	5.124996000	1.555047000	15.752887000
H	5.556702000	0.938114000	14.949781000
H	4.445760000	0.888112000	16.302464000
C	9.531413000	7.069978000	13.935976000
H	8.599840000	7.647923000	13.920749000
H	10.349088000	7.782503000	14.122465000
H	9.488033000	6.391050000	14.795061000
C	6.209308000	1.995370000	16.724239000
H	5.724033000	2.465639000	17.583749000
H	6.803837000	1.145448000	17.093287000
C	4.109448000	6.601181000	11.007236000
C	6.689187000	4.926712000	17.575652000
H	5.950388000	4.438642000	18.228862000
H	7.236683000	5.629611000	18.222481000
C	5.607093000	4.886224000	8.773192000
C	8.802943000	3.908442000	10.915139000

H	9.713303000	3.376065000	11.210395000
H	9.020974000	4.428077000	9.974784000
H	8.035456000	3.157548000	10.706694000
C	5.683047000	4.023838000	7.684659000
C	9.877123000	7.339899000	11.477627000
H	9.992170000	6.860266000	10.497742000
H	10.763513000	7.973891000	11.630134000
H	9.009242000	8.005662000	11.421276000
C	11.078811000	5.539809000	12.702473000
H	11.077631000	4.782439000	13.493544000
H	11.909686000	6.228803000	12.917290000
H	11.318949000	5.033988000	11.760580000
C	2.789251000	6.146920000	10.986249000
C	4.240473000	7.961440000	10.727328000
C	2.081702000	4.420741000	14.698817000
H	2.596708000	5.240517000	15.214284000
H	1.016211000	4.548365000	14.920678000
H	2.204334000	4.553116000	13.619673000
C	7.699706000	3.898691000	17.087884000
H	8.525126000	4.426523000	16.604224000
H	8.114786000	3.315895000	17.925219000
B	5.323528000	5.527908000	11.328259000
C	5.393627000	2.178340000	9.185184000
C	2.140922000	2.527115000	17.127290000
H	2.554818000	1.625266000	17.590905000
H	1.050531000	2.476218000	17.226064000
H	2.485070000	3.387377000	17.712421000
C	1.681071000	6.948724000	10.741490000
C	5.570343000	2.654947000	7.892931000
C	1.798500000	1.344784000	14.256697000
C	5.325603000	3.083472000	10.239322000
C	3.162169000	8.807178000	10.474269000
C	1.871094000	8.299341000	10.480583000
C	2.036968000	-0.053977000	14.847373000
H	3.093493000	-0.342434000	14.820530000
H	1.485085000	-0.808952000	14.267686000
H	1.693747000	-0.133160000	15.885656000
C	2.307117000	1.365268000	12.809645000
H	2.144260000	2.333542000	12.323606000
H	1.779232000	0.610958000	12.207229000
H	3.376018000	1.139009000	12.748243000
C	0.284978000	1.623290000	14.257010000
H	-0.137135000	1.643465000	15.268945000
H	-0.245470000	0.834713000	13.703272000

H	0.038984000	2.575807000	13.774377000
H	4.862561000	4.879661000	12.283396000
Si	5.647424000	7.314870000	16.706647000
C	5.006901000	7.943575000	15.029620000
H	5.835767000	8.146769000	14.342598000
H	4.447620000	8.879008000	15.139144000
H	4.328887000	7.228890000	14.543244000
C	7.206380000	8.291455000	17.179801000
H	7.509088000	8.132502000	18.220228000
H	7.034808000	9.366012000	17.049257000
H	8.050328000	8.012336000	16.540290000
C	2.946935000	7.071918000	17.614677000
H	2.595379000	7.517477000	16.676444000
H	2.172928000	7.264758000	18.372306000
H	2.996504000	5.986200000	17.474282000
C	4.295726000	7.651676000	18.058098000
C	4.677868000	7.040564000	19.414802000
H	4.709832000	5.946447000	19.381450000
H	3.935273000	7.317573000	20.177978000
H	5.652088000	7.392770000	19.774540000
C	4.151894000	9.173250000	18.232801000
H	5.072548000	9.635594000	18.606616000
H	3.358882000	9.397999000	18.961225000
H	3.880396000	9.678704000	17.298231000
H	7.145615000	6.706210000	10.799559000
H	6.653371000	7.000544000	12.433642000

3

U	5.695198000	10.027325000	3.301155000
Cl	4.691125000	12.628578000	3.162923000
Si	2.472913000	8.405103000	3.134494000
Si	7.447977000	10.717445000	0.183531000
Si	7.245243000	10.611823000	6.506026000
N	4.205994000	8.422012000	2.736607000
N	6.839624000	7.576429000	3.288025000
N	7.340356000	9.945045000	1.783034000
N	6.500546000	9.500242000	5.330599000

C	4.740650000	7.221189000	2.077237000
H	5.189232000	7.470577000	1.103592000
H	3.972792000	6.465730000	1.857547000
C	5.719624000	11.336357000	-0.280514000
H	5.045121000	10.486393000	-0.437582000
H	5.752960000	11.896858000	-1.221998000
H	5.277190000	11.991363000	0.476600000
C	5.792251000	6.583949000	2.971722000
H	5.310427000	6.282728000	3.906675000
H	6.234243000	5.682115000	2.518605000
C	2.203751000	9.804692000	4.381719000
H	2.661077000	9.561188000	5.346693000
H	1.138518000	9.977823000	4.566128000
H	2.626895000	10.753695000	4.031562000
C	6.075511000	11.012919000	7.997955000
C	2.075762000	6.716609000	3.986218000
H	2.413718000	5.912347000	3.322587000
H	2.794673000	6.767628000	4.819732000
C	1.358379000	8.736294000	1.581824000
C	7.888928000	7.603068000	2.247440000
H	7.436497000	7.314728000	1.293774000
H	8.681441000	6.872289000	2.474188000
C	7.679582000	12.210406000	5.584883000
H	8.524105000	12.036070000	4.907618000
H	7.987138000	12.992905000	6.288231000
H	6.847376000	12.612741000	4.997216000
C	8.438503000	9.014730000	2.107455000
H	8.945905000	9.306671000	3.039609000

H	9.223054000	9.003780000	1.338587000
C	-0.274780000	5.306242000	4.034907000
C	6.581622000	8.070991000	5.676996000
H	5.581662000	7.615650000	5.733939000
H	7.040617000	7.894171000	6.660016000
C	7.415619000	7.349493000	4.629027000
H	8.423993000	7.774256000	4.643768000
H	7.505032000	6.271676000	4.838196000
C	0.215098000	7.239252000	5.884055000
C	4.896681000	11.870037000	7.516771000
H	5.226414000	12.832692000	7.109603000
H	4.208286000	12.086518000	8.347343000
H	4.311326000	11.365936000	6.738214000
C	8.823619000	12.714812000	-1.300936000
H	7.857622000	13.034777000	-1.709302000
H	9.240167000	11.961567000	-1.979933000
H	9.492708000	13.587443000	-1.338947000
C	0.694055000	8.123320000	8.116485000
H	1.381652000	8.227120000	8.952340000
C	8.702650000	12.195664000	0.140791000
C	1.082583000	7.415897000	6.979465000
H	2.077833000	6.976699000	6.951700000
C	-1.064303000	7.822077000	5.981899000
H	-1.767311000	7.707814000	5.159800000
C	-2.146605000	3.751349000	4.333647000
H	-2.955257000	3.370854000	4.952801000
C	-0.056283000	4.714486000	2.773368000
H	0.762909000	5.068401000	2.152813000

C	-1.340684000	4.788907000	4.799880000
H	-1.534276000	5.199441000	5.787159000
C	8.868484000	9.879265000	7.174695000
H	8.721760000	9.049384000	7.873945000
H	9.434911000	10.655585000	7.701819000
H	9.499300000	9.520246000	6.353546000
C	1.625404000	10.167313000	1.090315000
H	1.354144000	10.919461000	1.839471000
H	1.032867000	10.378633000	0.187895000
H	2.678535000	10.323598000	0.829336000
C	1.668845000	7.759838000	0.439257000
H	2.706095000	7.847700000	0.097170000
H	1.023706000	7.967807000	-0.427447000
H	1.496265000	6.714797000	0.721573000
C	-1.914419000	3.202848000	3.072482000
H	-2.543417000	2.396887000	2.702481000
C	7.949160000	9.437518000	-1.132258000
H	8.980490000	9.081599000	-1.038227000
H	7.843537000	9.872544000	-2.132880000
H	7.287521000	8.564794000	-1.088476000
C	-1.449306000	8.559507000	7.101527000
H	-2.437159000	9.012336000	7.138801000
C	-0.572302000	8.706313000	8.176752000
H	-0.873752000	9.269769000	9.056238000
C	-0.864695000	3.688775000	2.290186000
H	-0.675785000	3.262722000	1.307927000
C	8.195194000	13.332452000	1.038972000
H	8.908350000	14.170427000	1.039965000

H	8.073040000	13.009333000	2.079316000
H	7.230710000	13.727426000	0.700137000
C	10.091538000	11.757871000	0.628588000
H	10.508618000	10.944234000	0.022701000
H	10.074435000	11.424445000	1.672521000
H	10.801181000	12.596965000	0.570984000
C	-0.122894000	8.615953000	1.965508000
H	-0.389993000	7.600303000	2.277202000
H	-0.763303000	8.864301000	1.105984000
H	-0.397388000	9.299669000	2.777364000
C	6.858757000	11.788890000	9.068924000
H	7.683716000	11.198578000	9.483974000
H	6.199661000	12.056443000	9.908253000
H	7.280823000	12.724399000	8.682186000
C	5.534326000	9.717055000	8.618902000
H	4.920880000	9.149040000	7.910152000
H	4.899937000	9.941914000	9.489385000
H	6.337697000	9.057625000	8.969612000
B	0.667042000	6.436230000	4.602269000

Int1

U	8.870582000	6.448759000	11.085478000
Si	12.299555000	5.746726000	10.308239000
Si	9.132626000	7.608014000	14.564587000
Cl	8.205222000	9.122650000	10.787344000
O	9.785252000	10.999618000	7.166071000
N	10.690534000	5.114502000	10.714840000

N	8.668161000	6.378351000	13.347261000
N	8.317220000	4.057806000	11.802043000
C	10.281686000	10.108500000	7.668745000
C	13.045225000	4.960347000	8.714429000
H	14.084013000	5.321008000	8.783178000
H	13.076295000	3.878428000	8.893339000
C	12.812345000	6.700568000	6.630156000
C	8.564609000	3.924696000	13.250736000
H	8.075403000	3.019282000	13.644556000
H	9.640975000	3.818449000	13.406169000
C	10.626258000	3.680513000	11.016043000
H	11.079896000	3.455388000	11.994100000
H	11.186738000	3.069040000	10.290676000
C	12.082070000	7.618107000	10.094321000
H	11.296677000	7.865277000	9.371779000
H	13.000939000	8.073049000	9.710724000
H	11.849514000	8.120036000	11.040390000
N	7.230322000	5.573562000	9.773939000
C	8.093201000	5.175692000	13.974644000
H	6.993159000	5.211950000	13.987678000
H	8.389504000	5.064767000	15.027150000
C	11.732332000	4.213653000	6.449746000
C	9.187442000	3.175845000	10.999526000
H	8.818897000	3.198423000	9.970534000
H	9.128915000	2.136184000	11.359771000
C	13.624443000	5.434104000	11.705064000
C	11.871969000	7.336866000	5.794128000
H	10.923003000	6.846584000	5.591324000

C	11.796432000	4.195460000	5.041155000
H	12.342942000	4.979569000	4.523672000
B	12.505312000	5.300359000	7.289157000
C	14.026918000	7.380097000	6.853042000
H	14.788539000	6.924618000	7.481903000
C	6.884591000	3.863946000	11.511517000
H	6.324073000	4.468562000	12.230652000
H	6.593625000	2.812847000	11.667719000
C	10.213679000	8.944366000	13.766926000
H	11.071264000	8.503348000	13.245743000
H	10.624002000	9.591817000	14.550597000
H	9.669308000	9.571691000	13.056999000
C	6.538551000	4.324082000	10.105503000
H	5.441684000	4.421323000	10.069330000
H	6.786519000	3.525873000	9.389460000
C	14.143575000	3.988243000	11.696353000
H	13.350498000	3.255002000	11.878186000
H	14.896197000	3.850830000	12.487050000
H	14.624723000	3.727859000	10.747091000
C	10.496457000	2.163062000	4.932255000
H	10.024231000	1.374944000	4.350935000
C	12.119412000	8.586314000	5.225588000
H	11.367855000	9.054491000	4.594669000
C	11.198668000	3.183447000	4.290966000
H	11.279103000	3.190919000	3.206715000
C	10.205860000	6.802418000	15.917544000
H	9.674495000	6.071016000	16.534456000
H	10.594417000	7.572952000	16.593990000

H	11.069347000	6.296686000	15.471191000
C	13.336272000	9.228219000	5.458412000
H	13.539176000	10.196356000	5.007235000
C	13.000967000	5.739210000	13.073528000
H	12.674038000	6.782753000	13.147351000
H	13.731682000	5.574273000	13.879358000
H	12.132456000	5.104441000	13.279578000
C	11.028697000	3.159303000	7.066475000
H	10.962218000	3.129850000	8.150510000
C	6.875812000	7.464408000	16.351481000
H	6.441352000	6.622473000	15.801594000
H	6.048456000	7.970165000	16.871968000
H	7.536860000	7.053142000	17.123195000
C	14.294294000	8.617195000	6.269107000
H	15.247811000	9.108155000	6.448065000
C	14.818157000	6.378595000	11.487686000
H	15.306275000	6.223251000	10.517610000
H	15.583462000	6.208620000	12.259565000
H	14.527572000	7.433106000	11.547749000
C	10.408282000	2.155507000	6.325903000
H	9.865198000	1.361206000	6.832086000
C	7.605227000	8.450348000	15.426135000
C	8.109338000	9.631187000	16.274134000
H	8.835702000	9.319603000	17.034599000
H	7.270187000	10.103412000	16.806765000
H	8.579848000	10.408302000	15.661320000
C	6.616845000	8.979391000	14.379042000
H	7.081492000	9.707230000	13.705411000

H	5.767438000	9.479857000	14.868737000
H	6.208226000	8.174245000	13.757893000
Si	6.568456000	6.371224000	8.310594000
C	5.042647000	7.396144000	8.791626000
H	4.299263000	6.790140000	9.321958000
H	4.550290000	7.840364000	7.918994000
H	5.340915000	8.213154000	9.458107000
C	7.170589000	4.161725000	6.554016000
H	8.113353000	4.665973000	6.315631000
H	6.905390000	3.546592000	5.681027000
H	7.365516000	3.474037000	7.383870000
C	4.746659000	4.402148000	7.167874000
H	4.841398000	3.705662000	8.006161000
H	4.447252000	3.808902000	6.290710000
H	3.916668000	5.082269000	7.392561000
C	6.049889000	5.160838000	6.871840000
C	7.903087000	7.519482000	7.606485000
H	8.290120000	8.189581000	8.378947000
H	7.479909000	8.154733000	6.820711000
H	8.737425000	6.956944000	7.173223000
C	5.810635000	6.025053000	5.618413000
H	5.052450000	6.800905000	5.779729000
H	5.452733000	5.395178000	4.790514000
H	6.724795000	6.519950000	5.273979000

TS1

U	8.728892000	6.507580000	11.121055000
Si	12.172825000	5.956368000	10.137939000
Si	9.059280000	7.594008000	14.622745000
Cl	8.157407000	9.189982000	10.863040000
O	10.080075000	7.396932000	6.397112000
N	10.568652000	5.276719000	10.531434000
N	8.678256000	6.350068000	13.385046000
N	8.354062000	4.067873000	11.772721000
C	10.851280000	6.777561000	6.958786000
C	12.926302000	5.154716000	8.569553000
H	14.010391000	5.209782000	8.753366000
H	12.686839000	4.086668000	8.658094000
C	13.574867000	6.828816000	6.471865000
C	8.782183000	3.897774000	13.173046000
H	8.421304000	2.937220000	13.574545000
H	9.874625000	3.884473000	13.197082000
C	10.568846000	3.819014000	10.717520000
H	11.138966000	3.530689000	11.613852000
H	11.050562000	3.286997000	9.882084000
C	11.908595000	7.829176000	9.966270000
H	11.044742000	8.079578000	9.340307000
H	12.777480000	8.306613000	9.501647000
H	11.758885000	8.310720000	10.939060000
N	6.949639000	5.637151000	9.969657000
C	8.295517000	5.069843000	14.010266000
H	7.207498000	4.999467000	14.158880000
H	8.731349000	4.946106000	15.011250000

C	12.149413000	4.514456000	6.053291000
C	9.154530000	3.265658000	10.829448000
H	8.670365000	3.330040000	9.851190000
H	9.169816000	2.205655000	11.129640000
C	13.485459000	5.660906000	11.555688000
C	13.327383000	7.355612000	5.187217000
H	12.521127000	6.940496000	4.586286000
C	12.854261000	4.162771000	4.887885000
H	13.762139000	4.701613000	4.628480000
B	12.720489000	5.613792000	7.066145000
C	14.631358000	7.412842000	7.195844000
H	14.869385000	7.044156000	8.189591000
C	6.908448000	3.817005000	11.641133000
H	6.407706000	4.362952000	12.445882000
H	6.682555000	2.746945000	11.775518000
C	10.078233000	9.003277000	13.870924000
H	10.947389000	8.613606000	13.328773000
H	10.467725000	9.632942000	14.679245000
H	9.508486000	9.637371000	13.187970000
C	6.384409000	4.323127000	10.309444000
H	5.286555000	4.344442000	10.397382000
H	6.598076000	3.581637000	9.524829000
C	14.032247000	4.224367000	11.559031000
H	13.258643000	3.478589000	11.769665000
H	14.802938000	4.116353000	12.336913000
H	14.500000000	3.953996000	10.606063000
C	11.267363000	2.414800000	4.364963000
H	10.927473000	1.611551000	3.715950000

C	14.082826000	8.399515000	4.656936000
H	13.858052000	8.781810000	3.664260000
C	12.429341000	3.121644000	4.060016000
H	13.004189000	2.867908000	3.172415000
C	10.162935000	6.833837000	15.978969000
H	9.645152000	6.138325000	16.646597000
H	10.576369000	7.633847000	16.604374000
H	11.010875000	6.301008000	15.533592000
C	15.128223000	8.950264000	5.399289000
H	15.724061000	9.761610000	4.988789000
C	12.856595000	5.957117000	12.922397000
H	12.522211000	6.997871000	13.000585000
H	13.585438000	5.793853000	13.730373000
H	11.992187000	5.314764000	13.122330000
C	10.989394000	3.770316000	6.344615000
H	10.419347000	4.005222000	7.242716000
C	6.780700000	7.297609000	16.360376000
H	6.411938000	6.439889000	15.786835000
H	5.910564000	7.745489000	16.863637000
H	7.442667000	6.913071000	17.144958000
C	15.402193000	8.450561000	6.672189000
H	16.215799000	8.870262000	7.258775000
C	14.668609000	6.619929000	11.341363000
H	15.179002000	6.446040000	10.386091000
H	15.420547000	6.479697000	12.132340000
H	14.363089000	7.671776000	11.368840000
C	10.543583000	2.745689000	5.512810000
H	9.635393000	2.200800000	5.759284000

C	7.475787000	8.337763000	15.468751000
C	7.900369000	9.527921000	16.346322000
H	8.622265000	9.238508000	17.119745000
H	7.026797000	9.950849000	16.864615000
H	8.348175000	10.336028000	15.756838000
C	6.480887000	8.830411000	14.410090000
H	6.916574000	9.595113000	13.758276000
H	5.594388000	9.272585000	14.889661000
H	6.130942000	8.014241000	13.767216000
Si	5.934423000	6.434426000	8.719895000
C	4.408533000	7.186266000	9.569289000
H	3.877047000	6.454085000	10.187696000
H	3.693113000	7.591279000	8.844425000
H	4.714099000	8.010956000	10.223854000
C	6.485827000	4.453955000	6.692912000
H	7.282033000	5.109412000	6.320713000
H	6.144595000	3.849269000	5.839243000
H	6.936542000	3.764741000	7.415969000
C	4.210632000	4.274725000	7.736764000
H	4.567988000	3.509017000	8.431589000
H	3.801705000	3.746380000	6.862336000
H	3.373243000	4.795504000	8.216402000
C	5.316796000	5.246695000	7.294990000
C	6.895030000	7.815256000	7.848591000
H	7.532135000	8.383007000	8.531008000
H	6.184937000	8.528373000	7.413590000
H	7.511393000	7.425897000	7.033242000
C	4.720566000	6.143298000	6.192018000

H	3.881090000	6.748387000	6.555114000
H	4.333858000	5.520576000	5.371792000
H	5.458698000	6.825182000	5.757226000

Int2

U	-0.904319000	-0.286613000	0.171480000
Si	2.581552000	-1.560582000	-0.462000000
Si	-2.331891000	2.964804000	1.189203000
Cl	-0.495375000	0.571302000	-2.427695000
O	1.987204000	2.632299000	0.100071000
N	1.262887000	-0.857129000	0.536475000
N	-1.874046000	1.273228000	1.522881000
N	-0.652367000	-1.031847000	2.702748000
C	2.731137000	1.921441000	-0.391831000
C	3.212372000	-0.283423000	-1.720630000
H	4.013765000	-0.749635000	-2.311311000
H	2.382404000	-0.112578000	-2.417611000
C	5.163701000	1.033012000	-0.282903000
C	-1.026936000	0.128824000	3.535084000
H	-1.254143000	-0.190790000	4.564633000
H	-0.167312000	0.805012000	3.579978000
C	1.620187000	-0.552267000	1.937691000
H	1.466819000	0.516707000	2.164610000
H	2.677830000	-0.738607000	2.154667000
C	4.015557000	-2.136488000	0.649949000
H	4.543619000	-1.310020000	1.133104000
H	4.752107000	-2.659455000	0.028170000

H	3.690851000	-2.835858000	1.428224000
N	-2.130663000	-2.131379000	0.567597000
C	-2.191676000	0.876617000	2.908929000
H	-3.097164000	0.251967000	2.947715000
H	-2.402865000	1.738771000	3.558680000
C	3.998100000	2.298621000	-2.387753000
C	0.773062000	-1.380201000	2.892542000
H	0.907714000	-2.437809000	2.647985000
H	1.067815000	-1.235611000	3.943637000
C	2.001848000	-3.144205000	-1.431241000
C	5.278099000	1.462617000	1.049068000
H	4.424184000	1.908529000	1.557884000
C	4.753892000	3.459823000	-2.145342000
H	5.221887000	3.599426000	-1.172893000
B	3.829344000	1.141694000	-1.209166000
C	6.311062000	0.471961000	-0.872515000
H	6.271857000	0.138830000	-1.907836000
C	-1.543795000	-2.186145000	2.944908000
H	-2.527427000	-1.801266000	3.227581000
H	-1.169417000	-2.795458000	3.782002000
C	-1.717762000	3.461375000	-0.532673000
H	-0.644626000	3.284380000	-0.648665000
H	-1.880872000	4.535974000	-0.674870000
H	-2.231193000	2.940008000	-1.345757000
C	-1.692973000	-3.002070000	1.669795000
H	-2.395476000	-3.821878000	1.871153000
H	-0.734868000	-3.490880000	1.434705000
C	0.669295000	-2.910803000	-2.149290000

H	-0.143559000	-2.700196000	-1.441446000
H	0.372353000	-3.808365000	-2.712999000
H	0.716717000	-2.077566000	-2.857376000
C	4.350003000	4.271297000	-4.384841000
H	4.487598000	5.026752000	-5.154477000
C	6.471300000	1.330720000	1.764865000
H	6.522728000	1.673332000	2.795852000
C	4.933296000	4.433134000	-3.127237000
H	5.528675000	5.317253000	-2.911443000
C	-1.473373000	4.119372000	2.435049000
H	-1.715059000	3.901481000	3.480401000
H	-1.747045000	5.163619000	2.246023000
H	-0.385183000	4.044910000	2.324856000
C	7.590700000	0.763523000	1.159504000
H	8.520953000	0.658311000	1.712047000
C	1.846104000	-4.319895000	-0.455299000
H	2.801876000	-4.610939000	-0.005242000
H	1.451244000	-5.204647000	-0.976650000
H	1.153177000	-4.089527000	0.362691000
C	3.408629000	2.165618000	-3.654663000
H	2.809283000	1.288873000	-3.882851000
C	-4.830719000	3.113790000	2.658420000
H	-4.743013000	2.085631000	3.025014000
H	-5.902503000	3.363538000	2.660672000
H	-4.347044000	3.772836000	3.389419000
C	7.505235000	0.335155000	-0.166749000
H	8.372406000	-0.105888000	-0.653087000
C	3.062275000	-3.519182000	-2.480354000

H	3.153930000	-2.759557000	-3.264549000
H	2.790054000	-4.463271000	-2.975673000
H	4.056189000	-3.664810000	-2.040119000
C	3.588628000	3.132354000	-4.645650000
H	3.129985000	2.994926000	-5.622191000
C	-4.249872000	3.283638000	1.246343000
C	-4.524306000	4.724680000	0.781241000
H	-4.032556000	5.469834000	1.418030000
H	-5.603537000	4.936542000	0.814536000
H	-4.195035000	4.897994000	-0.249268000
C	-4.960029000	2.313037000	0.293082000
H	-4.609330000	2.421169000	-0.740410000
H	-6.045046000	2.494977000	0.286224000
H	-4.806380000	1.269862000	0.591601000
Si	-3.483242000	-2.720558000	-0.422758000
C	-3.819847000	-1.373072000	-1.717650000
H	-4.316485000	-0.491960000	-1.296441000
H	-4.465515000	-1.764867000	-2.511813000
H	-2.895740000	-1.039480000	-2.206224000
C	-5.110652000	-4.405068000	1.301900000
H	-4.897028000	-5.247365000	0.634042000
H	-6.090740000	-4.600151000	1.762807000
H	-4.371221000	-4.426021000	2.109786000
C	-5.431477000	-1.935570000	1.553542000
H	-4.677625000	-1.882404000	2.346320000
H	-6.405806000	-2.093835000	2.039881000
H	-5.471837000	-0.953754000	1.067115000
C	-5.135150000	-3.061199000	0.555418000

C	-3.005583000	-4.318534000	-1.334158000
H	-2.252092000	-4.103490000	-2.099693000
H	-3.867868000	-4.769567000	-1.838706000
H	-2.584199000	-5.070055000	-0.657533000
C	-6.277123000	-3.114715000	-0.476705000
H	-6.429581000	-2.151995000	-0.976158000
H	-7.223431000	-3.375767000	0.019866000
H	-6.106966000	-3.869913000	-1.253867000

TS2

U	9.269361000	5.366060000	11.855680000
Si	12.793459000	3.933536000	11.307769000
Si	7.820829000	8.556759000	12.880490000
Cl	9.824713000	6.155879000	9.275552000
O	12.092184000	7.798640000	12.195322000
N	11.588039000	4.934468000	12.231298000
N	8.270407000	6.856547000	13.222149000
N	9.513988000	4.550095000	14.325642000
C	12.616143000	6.985131000	11.569021000
C	13.426846000	5.069678000	9.943091000
H	14.300618000	4.621869000	9.454711000
H	12.638045000	5.128217000	9.182465000
C	15.176544000	6.605886000	11.385577000
C	9.161631000	5.688715000	15.200526000
H	8.946592000	5.336018000	16.221121000
H	10.026137000	6.356714000	15.255963000
C	11.835290000	5.065404000	13.681667000

H	11.664473000	6.107931000	13.994050000
H	12.875796000	4.852135000	13.952299000
C	14.214633000	3.408503000	12.457462000
H	14.752774000	4.263740000	12.875034000
H	14.939864000	2.834232000	11.868240000
H	13.891212000	2.769985000	13.286369000
N	7.972122000	3.578581000	12.178542000
C	7.991318000	6.461952000	14.615850000
H	7.075242000	5.855672000	14.676952000
H	7.818547000	7.325411000	15.274265000
C	13.746633000	7.779692000	9.439301000
C	10.931023000	4.159958000	14.507774000
H	11.049991000	3.126288000	14.167537000
H	11.199546000	4.191994000	15.575298000
C	12.104483000	2.291520000	10.530215000
C	15.341687000	7.120858000	12.682115000
H	14.485400000	7.501237000	13.235662000
C	14.031472000	9.101908000	9.825731000
H	14.245705000	9.320774000	10.870726000
B	13.780230000	6.538791000	10.521770000
C	16.341312000	6.146926000	10.740548000
H	16.270551000	5.753356000	9.728672000
C	8.598498000	3.405760000	14.539405000
H	7.631109000	3.800690000	14.860177000
H	8.977633000	2.754972000	15.341438000
C	8.420050000	9.002866000	11.140161000
H	9.485175000	8.788302000	11.006819000
H	8.292464000	10.079902000	10.980915000

H	7.873963000	8.489007000	10.343217000
C	8.404062000	2.649810000	13.235264000
H	7.683896000	1.840702000	13.412781000
H	9.343241000	2.146481000	12.959653000
C	10.770948000	2.515894000	9.809952000
H	9.962448000	2.775559000	10.505210000
H	10.453092000	1.596316000	9.295755000
H	10.832142000	3.307149000	9.055398000
C	13.817820000	9.900618000	7.554927000
H	13.847198000	10.712360000	6.832034000
C	16.590572000	7.164754000	13.309276000
H	16.673266000	7.567189000	14.316581000
C	14.063651000	10.149416000	8.906758000
H	14.287694000	11.159442000	9.243054000
C	8.718314000	9.703034000	14.103062000
H	8.468981000	9.509635000	15.151369000
H	8.473952000	10.751076000	13.895802000
H	9.803319000	9.594909000	13.995050000
C	17.723804000	6.696891000	12.648383000
H	18.697072000	6.728816000	13.131747000
C	11.938739000	1.196685000	11.593805000
H	12.899086000	0.903939000	12.032483000
H	11.496052000	0.291677000	11.151795000
H	11.284358000	1.504755000	12.417902000
C	13.501285000	7.558887000	8.075202000
H	13.282029000	6.552991000	7.726286000
C	5.361915000	8.696436000	14.402347000
H	5.434846000	7.654462000	14.731218000

H	4.296627000	8.969583000	14.438928000
H	5.877572000	9.319233000	15.143101000
C	17.592096000	6.190041000	11.354032000
H	18.467531000	5.826934000	10.819922000
C	13.131759000	1.799719000	9.492765000
H	13.228120000	2.490022000	8.647784000
H	12.819550000	0.827104000	9.084489000
H	14.130460000	1.660274000	9.924042000
C	13.541099000	8.598368000	7.142332000
H	13.354170000	8.387405000	6.091529000
C	5.912062000	8.900897000	12.982475000
C	5.655618000	10.360390000	12.568046000
H	6.157092000	11.075200000	13.230995000
H	4.579078000	10.583295000	12.609914000
H	5.986167000	10.566123000	11.543863000
C	5.162847000	7.972813000	12.017544000
H	5.471011000	8.127258000	10.976754000
H	4.078963000	8.155730000	12.062446000
H	5.325953000	6.917628000	12.264166000
Si	6.599163000	3.064103000	11.163909000
C	6.285017000	4.509179000	9.977749000
H	5.835655000	5.376488000	10.473294000
H	5.609843000	4.203975000	9.170691000
H	7.214310000	4.838927000	9.494995000
C	4.977372000	1.280532000	12.784588000
H	5.188439000	0.487784000	12.058000000
H	3.995128000	1.058220000	13.227712000
H	5.713508000	1.196854000	13.591650000

C	4.694257000	3.728510000	13.222474000
H	5.464021000	3.716781000	14.001422000
H	3.727855000	3.542784000	13.714446000
H	4.654122000	4.742956000	12.809584000
C	4.960622000	2.676264000	12.139879000
C	7.054457000	1.536059000	10.131306000
H	7.752434000	1.816671000	9.334732000
H	6.170790000	1.098621000	9.652578000
H	7.529992000	0.753527000	10.732332000
C	3.807178000	2.712356000	11.119235000
H	3.665224000	3.709075000	10.687820000
H	2.862470000	2.432724000	11.608307000
H	3.957472000	2.008924000	10.291034000

Int3

U	-1.191237000	-0.038651000	-0.391390000
Si	4.230480000	-2.055604000	-0.012715000
Si	-1.672453000	2.869784000	1.603831000
Cl	-1.007916000	-0.277772000	-3.095091000
O	1.050034000	0.176835000	-0.142449000
N	2.594477000	-1.349258000	0.458083000
N	-1.683769000	1.093123000	1.460511000
N	-0.705167000	-1.620423000	1.666176000
C	2.228137000	-0.322300000	-0.323719000
C	4.441253000	-0.998913000	-1.531969000
H	5.481766000	-0.698486000	-1.697694000
H	4.143849000	-1.600395000	-2.401184000

C	4.189401000	1.436070000	-0.391273000
C	-0.824077000	-0.754901000	2.866584000
H	-1.018229000	-1.368144000	3.760767000
H	0.125610000	-0.238786000	3.015961000
C	1.891651000	-1.630070000	1.712566000
H	1.771165000	-0.683924000	2.247519000
H	2.575013000	-2.230510000	2.318099000
C	5.420533000	-1.737485000	1.418681000
H	5.497059000	-0.663104000	1.612743000
H	6.423149000	-2.100995000	1.167233000
H	5.113639000	-2.232209000	2.346002000
N	-2.776670000	-1.563661000	-0.333977000
C	-1.899587000	0.313299000	2.690359000
H	-2.898335000	-0.146793000	2.693159000
H	-1.858703000	0.937706000	3.594056000
C	2.859757000	0.974191000	-2.667351000
C	0.566924000	-2.399399000	1.667513000
H	0.553163000	-3.052895000	0.789370000
H	0.544003000	-3.054712000	2.550887000
C	4.059422000	-3.947199000	-0.341028000
C	3.807310000	1.867448000	0.891407000
H	2.929924000	1.432868000	1.367879000
C	2.233147000	2.236757000	-2.642133000
H	2.148878000	2.762422000	-1.692479000
B	3.419368000	0.288375000	-1.294019000
C	5.331680000	2.061581000	-0.932396000
H	5.666702000	1.776017000	-1.928750000
C	-1.818769000	-2.617501000	1.636250000

H	-2.659450000	-2.218597000	2.208334000
H	-1.499010000	-3.551181000	2.119783000
C	-1.244795000	3.532693000	-0.126618000
H	-0.273014000	3.163769000	-0.475416000
H	-1.166210000	4.625099000	-0.099334000
H	-2.000571000	3.288192000	-0.882656000
C	-2.279272000	-2.837295000	0.206349000
H	-3.035318000	-3.631849000	0.190381000
H	-1.438687000	-3.227907000	-0.394390000
C	2.915147000	-4.225317000	-1.325217000
H	1.939202000	-3.950013000	-0.909102000
H	2.874513000	-5.296109000	-1.571753000
H	3.036547000	-3.680320000	-2.268365000
C	1.859182000	2.221619000	-5.031781000
H	1.478832000	2.696590000	-5.933265000
C	4.513956000	2.848664000	1.595730000
H	4.179637000	3.149392000	2.586892000
C	1.734442000	2.851153000	-3.791655000
H	1.257100000	3.827016000	-3.722419000
C	-0.312337000	3.403965000	2.813194000
H	-0.420675000	2.941539000	3.800196000
H	-0.307686000	4.489833000	2.958291000
H	0.669248000	3.119943000	2.418058000
C	5.640323000	3.441889000	1.030297000
H	6.193461000	4.205999000	1.571383000
C	3.828091000	-4.743319000	0.950713000
H	4.632047000	-4.589173000	1.679412000
H	3.790289000	-5.820890000	0.733925000

H	2.881177000	-4.480809000	1.436725000
C	2.984524000	0.380220000	-3.935122000
H	3.480379000	-0.583743000	-4.028116000
C	-3.709509000	3.235310000	3.610033000
H	-3.882375000	2.158948000	3.716947000
H	-4.630886000	3.741854000	3.933506000
H	-2.921420000	3.520747000	4.316894000
C	6.046574000	3.040907000	-0.244846000
H	6.922216000	3.495513000	-0.704292000
C	5.387609000	-4.399529000	-0.976614000
H	5.596706000	-3.873221000	-1.915693000
H	5.352593000	-5.474153000	-1.206834000
H	6.243519000	-4.242057000	-0.309169000
C	2.496393000	0.983044000	-5.098123000
H	2.618544000	0.485103000	-6.058305000
C	-3.366619000	3.629694000	2.164658000
C	-3.265230000	5.163352000	2.088134000
H	-2.475316000	5.563637000	2.734444000
H	-4.210114000	5.620752000	2.416474000
H	-3.073785000	5.518481000	1.068993000
C	-4.494603000	3.161241000	1.235939000
H	-4.316809000	3.454210000	0.194240000
H	-5.454228000	3.607709000	1.535759000
H	-4.620416000	2.072981000	1.265312000
Si	-4.359188000	-1.545704000	-1.151556000
C	-4.572415000	0.208891000	-1.837264000
H	-4.649245000	0.968654000	-1.051950000
H	-5.485851000	0.272138000	-2.439265000

H	-3.740510000	0.469953000	-2.501447000
C	-5.829570000	-3.397311000	0.526343000
H	-5.782523000	-4.125712000	-0.291786000
H	-6.744566000	-3.614892000	1.097427000
H	-4.985762000	-3.590151000	1.198008000
C	-5.868864000	-0.997858000	1.225247000
H	-4.959813000	-1.094822000	1.829789000
H	-6.724585000	-1.217062000	1.880750000
H	-5.955773000	0.051720000	0.921959000
C	-5.856305000	-1.946625000	0.019724000
C	-4.347137000	-2.784644000	-2.586988000
H	-3.553865000	-2.522213000	-3.295436000
H	-5.298300000	-2.777645000	-3.130826000
H	-4.164737000	-3.810234000	-2.248005000
C	-7.152762000	-1.741439000	-0.784994000
H	-7.260134000	-0.712821000	-1.147255000
H	-8.027615000	-1.953692000	-0.152921000
H	-7.215148000	-2.408356000	-1.653031000

5

U	7.892352000	6.382337000	11.769831000
Si	13.199679000	3.734625000	12.075026000
Si	8.983230000	9.343134000	13.696013000
Cl	5.333570000	7.294154000	11.740617000
O	10.071078000	5.899963000	11.221103000
N	11.621587000	4.656417000	12.295429000
O	7.990277000	7.974463000	9.807640000

N	8.255339000	7.729761000	13.510151000
N	8.453449000	4.939522000	13.900503000
C	11.205818000	5.284976000	11.181744000
C	13.276313000	3.979867000	10.229972000
H	14.299335000	4.113388000	9.862043000
H	12.878111000	3.085657000	9.732273000
C	13.223690000	6.666780000	10.142136000
C	8.498048000	5.848225000	15.071222000
H	8.098719000	5.343611000	15.963737000
H	9.538119000	6.106948000	15.280175000
C	10.957470000	4.901640000	13.574623000
H	10.738709000	5.972675000	13.637243000
H	11.681127000	4.680309000	14.362736000
C	14.509257000	4.628854000	13.100801000
H	14.610017000	5.663015000	12.757154000
H	15.485916000	4.144970000	12.988528000
H	14.268819000	4.645376000	14.169023000
N	7.049076000	4.323598000	11.548699000
C	7.737199000	7.127694000	14.753181000
H	6.659845000	6.911971000	14.668147000
H	7.825525000	7.795635000	15.619387000
C	11.628841000	5.225312000	8.460399000
C	9.668242000	4.097914000	13.818112000
H	9.533828000	3.383599000	13.003751000
H	9.754308000	3.515485000	14.747939000
C	9.017556000	7.837116000	8.776999000
H	9.202581000	6.774481000	8.621085000
H	9.931823000	8.308274000	9.149562000

C	13.019645000	1.908587000	12.668269000
C	12.967106000	7.691599000	11.069756000
H	12.095428000	7.620580000	11.716779000
C	11.792228000	6.199642000	7.459022000
H	12.369603000	7.094391000	7.680114000
B	12.311265000	5.312408000	9.944316000
C	14.382444000	6.835920000	9.354923000
H	14.637842000	6.073829000	8.620252000
C	7.248927000	4.084155000	13.989207000
H	6.401176000	4.734412000	14.231486000
H	7.352978000	3.357285000	14.810800000
C	9.733835000	9.855231000	12.036111000
H	10.403508000	9.084335000	11.643697000
H	10.333708000	10.763318000	12.165051000
H	8.976671000	10.065315000	11.276370000
C	6.971575000	3.381553000	12.671175000
H	5.974621000	2.923781000	12.763753000
H	7.670424000	2.542142000	12.537604000
C	11.852878000	1.216271000	11.952363000
H	10.891229000	1.687914000	12.181629000
H	11.784795000	0.163165000	12.261428000
H	11.974875000	1.225784000	10.863551000
C	10.502038000	4.930223000	5.850726000
H	10.078229000	4.814804000	4.855883000
C	13.797495000	8.808802000	11.209102000
H	13.556763000	9.572828000	11.945576000
C	11.247175000	6.061612000	6.178623000
H	11.408880000	6.840430000	5.435650000

C	10.386876000	9.241160000	14.975435000
H	10.037684000	8.935827000	15.967912000
H	10.889993000	10.208015000	15.089292000
H	11.142900000	8.516866000	14.651095000
C	14.929130000	8.944403000	10.407851000
H	15.578287000	9.810882000	10.509269000
C	12.829288000	1.796978000	14.187838000
H	13.638233000	2.284181000	14.744365000
H	12.818600000	0.740373000	14.493146000
H	11.881728000	2.234839000	14.521372000
C	7.023656000	8.990867000	9.398827000
H	6.831093000	9.639993000	10.254122000
H	6.094563000	8.483572000	9.123867000
C	10.871195000	4.095875000	8.091711000
H	10.719343000	3.301234000	8.820841000
C	7.028239000	10.392234000	15.572792000
H	6.364823000	9.525848000	15.481918000
H	6.404590000	11.241194000	15.891125000
H	7.736120000	10.193864000	16.386873000
C	15.217143000	7.946775000	9.473871000
H	16.097823000	8.032814000	8.840353000
C	14.330914000	1.200484000	12.278101000
H	14.522282000	1.250282000	11.199326000
H	14.281924000	0.136337000	12.550632000
H	15.201488000	1.624613000	12.792156000
C	10.315002000	3.942867000	6.820617000
H	9.743364000	3.048278000	6.581726000
C	7.730953000	10.723471000	14.247121000

C	8.508843000	12.038992000	14.426348000
H	9.245426000	11.980742000	15.236043000
H	7.816657000	12.856162000	14.678270000
H	9.039113000	12.338720000	13.514743000
C	8.460617000	8.560481000	7.564237000
H	9.256263000	8.912695000	6.903529000
H	7.802196000	7.898009000	6.991676000
C	7.659606000	9.690872000	8.210447000
H	6.904646000	10.122744000	7.548079000
H	8.327606000	10.493150000	8.542763000
C	6.660566000	10.925243000	13.164689000
H	7.093110000	11.276171000	12.220555000
H	5.931998000	11.685401000	13.484802000
H	6.106323000	10.002537000	12.960556000
Si	6.087785000	3.797469000	10.130359000
C	4.234717000	3.854179000	10.536743000
H	3.984152000	3.294908000	11.444497000
H	3.639964000	3.436547000	9.715865000
H	3.924406000	4.892926000	10.687863000
C	8.038388000	1.797327000	9.468240000
H	8.524669000	2.538481000	8.826130000
H	8.280582000	0.803872000	9.061709000
H	8.500391000	1.865369000	10.459250000
C	5.891554000	0.925076000	10.418305000
H	6.290193000	0.938953000	11.437995000
H	6.099041000	-0.074105000	10.006759000
H	4.802235000	1.024657000	10.484896000
C	6.520426000	2.003951000	9.521545000

C	6.426775000	5.032458000	8.733257000
H	6.205521000	6.059515000	9.044407000
H	5.764970000	4.819582000	7.886282000
H	7.457191000	4.988919000	8.365908000
C	5.950899000	1.831477000	8.100696000
H	4.866993000	1.995408000	8.060776000
H	6.132800000	0.806958000	7.744053000
H	6.417553000	2.510894000	7.378833000

References

1. R. Boaretto, P. Roussel, N. W. Alcock, A. J. Kingsley, I. J. Munslow, C. J. Sanders, P. Scott, *J. Organomet. Chem.* **1999**, *591*, 174.

2. J. N. Bently, E. Pradhan, T. Zeng, C. B. Caputo, *Dalton trans.* **2020**, *49*, 16054.

3. H Braunschweig, Q Ye, K Radackia, *Chem. Commun*. **2012**, *48*, 2701.

4. a) M. Helliwell, E. J. Thomas, L. A. Townsend, *J. Chem. Soc. Perkin Trans.* **2002**, *1*, 1286; b) J. C. Thomas, J. C. Peters, I*norg. Chem.* **2003**, *42*, 5055.

5. J. Wang, P. Jia, W. Sun, U. Wei, Z. Lin, Q. Ye, *Inorg. Chem.* **2022,** *61*, 8879.

6 G. M. Sheldrick, *Acta Cryst.* **2015**, *C71*, 3.

7 O. V. Dolomanov, L. J. Bourhis, R. J. Gildea, J. A. K. Howard, H. Puschmann, *J. Appl. Cryst.* **2009**, *42*, 339.

8. (a) A. D. Becke, *J. Chem. Phys*. **1993**, *98*, 5648; (b) K. Burke, J. P. Perdew, W. Yang, *in Electronic Density Functional Theory: Recent Progress and New Directions,* Eds: J. F. Dobson, G. Vignale, M. P. Das, Plenum, New York, **1998**

9. (a) W. Kuechle, M. Dolg, H. Stoll, H. Preuss, *J. Chem. Phys*. **1994**, *100*, 7535; b) X. Cao, M. Dolg, H. Stoll, *J. Chem. Phys.* **2003**, *118*, 487; c) X. Cao, M. Dolg, *J. Molec. Struct. (Theochem)* **2004**, *673*, 203.

10. (a) P. C. Hariharan and J. A. Pople, *Theor. Chim. Acta* **1973**, *28*, 213; (b) W. J. Hehre, R. Ditchfield and J. A. Pople, *J. Chem. Phys.* **1972**, *56*, 2257.

11. A. V. Marenich, C. J. Cramer, and D. G. Truhlar, *J. Chem. Phys. B* **2009**, *113*, 6378.

12. Gaussian 09, Revision D.01: M. J. Frisch, G. W. Trucks, H. B. Schlegel, G. E. Scuseria, M. A. Robb, J. R. Cheesman, G. Scalmani, V. Barone, B. Mennucci, G. A. Petersson, H. Nakatsuji, M. Caricato, X. Li, H. P. Hratchian, A. F. Izmaylov, J. Bloino, G. Zheng, J. L. Sonnenberg, M. Hada, M. Ehara, K. Toyota, R. Fukuda, J. Hasegawa, M. Ishida, T. Nakajima, Y. Honda, O. Kitao, H. Nakai, T. Vreven, J. A., Jr. Montgomery, J. E. Peralta, F. Ogliaro, M. Bearpark, J. J. Heyd, E. Brothers, K. N. Kudin, V. N. Staroverov, R. Kobayashi, J. Normand, K. Raghavachari, J. C. Burant, S. S. Iyengar, J. Tomasi, M. Cossi, N. Rega, M. J. Millam, M. Klene, J. E. Knox, J. B. Cross, V. Bakken, C. Adamo, J. Jaramillo, R. Gomperts, R. E. Stratmann, O. Yazyev, A. J. Austin, R. Cammi, C. Pomelli, J. W. Ochterski, R. L. Martin, K. Morokuma, V. G. Zakrzewski, G. A. Voth, P. Salvador, J. J. Dannenberg, S. Dapprich, A. D. Daniels, O. Farkas, J. B. Foresman, J. V. Ortiz, J.

Cioslowski and D. J. Fox, Gaussian Inc., **2009**, Wallingford CT.
